# A Narrative Review of the Potential Impact of Next-Generation Tobacco and Nicotine Products on Cardiovascular Health

**DOI:** 10.7759/cureus.111861

**Published:** 2026-07-01

**Authors:** Ian M Fearon, Matthew Stevenson, Thomas Nahde

**Affiliations:** 1 Scientific Research, whatIF? Consulting Ltd, Harwell, GBR; 2 Group Science and Regulatory Affairs, Imperial Brands PLC, Bristol, GBR; 3 Group Science and Regulatory Affairs, Reemstma Cigarettenfabriken GmbH, Hamburg, DEU

**Keywords:** cardiovascular disease, cigarette smoking, electronic vaping products, heated tobacco products, nicotine pouches, tobacco harm reduction

## Abstract

Cigarette smoking is a cause of serious disease in smokers, including cardiovascular disease (CVD). The processes underpinning the development of CVD are well understood. These involve many cell types and cellular processes contributing to the development of atherosclerotic plaques in blood vessels, leading to impaired tissue oxygen supply and acute manifestations such as myocardial infarction and stroke. Cigarette smoke contains thousands of chemicals, some of which can be harmful to the cardiovascular system and may promote atherogenesis. In this narrative review, we assess the growing body of evidence concerning the potential cardiovascular health impact of next‑generation tobacco and nicotine product (NGP) use, including electronic vaping product (EVP), heated tobacco product (HTP) and oral nicotine pouch (ONP) use. Exclusive use of NGPs substantially reduces exposure to harmful toxicants compared with cigarette smoking. Regarding CVD risk, we determine that: (i) in vivo and in vitro* *modelling studies demonstrate the potential for NGPs to reduce CVD risk compared with cigarette smoking; (ii) switching to the exclusive use of these products leads to reductions in biomarkers of exposure to cardiovascular toxicants and favourable changes in CVD risk-related biomarkers of potential harm; (iii) population‑level data show that switching to the exclusive use of both EVPs and HTPs is associated with reduced risk of CVD and related comorbidities compared with continued smoking, though data for ONPs in this area are lacking; and (iv) regulators are increasingly using science‑based evidence when approving marketing authorisations and the use of consumer‑facing reduced exposure claims. Overall, supporting the complete displacement of cigarettes with the use of NGPs has the potential to reduce CVD risk and improve both individual and population health.

## Introduction and background

Smoking and cardiovascular disease

Cigarette smoking is the leading preventable cause of death and disease and is associated with the development of cardiovascular disease (CVD), chronic lung disease, and cancer [1]. Many other risk factors also increase the propensity for CVD development, including poor diet and obesity, lack of physical activity, alcohol consumption, diabetes, hypertension, dyslipidaemia and mental health [2]. Smoking is reported to be responsible for one in every four deaths in the United States (US) from CVD [3], and smokers are reportedly two to five times more likely to experience both fatal and non‑fatal CVD events than non‑smokers [4,5]. This elevated risk causes a reported 2 million smoking‑attributable deaths globally each year from ischaemic heart disease [1,6], a principal manifestation of CVD. While the level of cigarette consumption may influence the degree of CVD risk [1], even low levels of cigarette smoking significantly elevate the likelihood of the development of CVD [4,7]. Quitting smoking generates significant improvements in cardiovascular health, and over time, the risk of cardiovascular‑related mortality among smokers who have quit smoking becomes comparable to that of never-smokers [7-10]. 

Multifactorial processes underpin cardiovascular disease development

**Figure 1:**
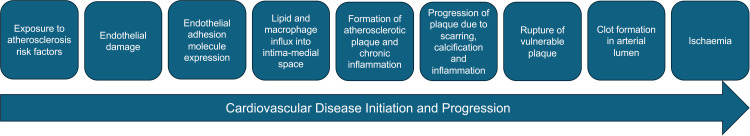
. Sequence of Events in the Initiation and Progression of Atherosclerotic Cardiovascular Disease. Image credits: Ian M. Fearon

Materialising in many forms, cardiovascular disease may be broadly classified into coronary heart disease, cerebrovascular disease, and peripheral vascular disease, in which the blood supplies to the heart, the brain, and the peripheral vasculature, respectively, are compromised. Common to each of these classifications is the formation of an atherosclerotic plaque or lesion (an atheroma), which occludes blood vessels and disrupts blood flow. This leads to acute manifestations such as myocardial infarction (MI) and stroke, in which tissue oxygen and nutrient supply are severely compromised. A complication of atherosclerotic plaques is their vulnerability to rupture, giving rise to a thrombus that has the ability to occlude vessels away from the initial atheroma site [11-13]. Regardless of the clinical manifestation, the biological mechanisms underpinning the initiation of atherosclerotic CVD are well understood. Although initially considered to be a simple disease involving arterial lipid accumulation, atherosclerosis is now known to involve a well‑defined cascade of inflammatory processes (Figure 1) [12,13].

The initiating step in the development of an atherosclerotic lesion is damage to the endothelium [11,14,15], a single layer of cells lining blood vessels, which is a principal regulator of vascular function. In a healthy individual and prior to the onset of cardiovascular disease, the endothelium plays a homeostatic role in maintaining vascular tone and blood flow [14]. In the early stages of cardiovascular disease progression, endothelial dysfunction triggers a chronic inflammatory process in the vessel wall. Induction of adhesion molecules in endothelial cells facilitates the attachment of monocytic white blood cells (WBCs), trapping them and allowing their transmigration through the endothelial layer into the underlying intimal layer. Here, they become tissue macrophages and subsequently foam cells, following the uptake of oxidatively modified lipids [11,16].

Other cell types become involved in the enlargement of the atherosclerotic lesion, including vascular smooth muscle cells (VSMCs). Whether already residing in the intimal layer or following their migration into this layer from the medial layer, these cells begin proliferating and contribute to plaque growth [11,17]. Platelets are also recruited to the lesion site due to the release of chemical attractants from the injured intimal layer [18,19]. Altered vasoreactivity and enhanced propensity for clot formation, due to the loss of anti-thrombotic factors and an increase in pro-thrombotic factors, contribute to plaque formation and disease progression. 

A pivotal factor in CVD initiation and progression is oxidative stress [20]. Reactive oxygen species (ROS), also termed oxygen free radicals, such as superoxide, peroxides, hydroxyl radicals, and hypochlorous acid, are an integral part of numerous physiological cellular signalling pathways in many cell types within the cardiovascular system. However, ROS are also highly reactive and damaging to cells. Oxidative stress occurs when an imbalance develops between the production of ROS and the capacity of the cellular antioxidant defence, leading to an altered redox status that can contribute to endothelial dysfunction [21-23]. Many types of ROS have also been implicated in the migration of smooth muscle cells into the intimal layer [17]. This may be facilitated by ROS-dependent alterations in the expression and activation of matrix metalloproteinases [24], which degrade the intimal layer and promote smooth muscle migration. Proliferation of vascular smooth muscle cells is also ROS-regulated. Although the exact type of ROS involved and the direction of the proliferative response (increased or decreased) are complex and unclear [17]. Monocytes/macrophages also possess the ability to produce ROS, which play a role in lesion progression and inflammation and have been shown to cause oxidative modification of the low‑density lipoprotein (LDL) [25,26]. Platelets too can produce, and be activated by, superoxide and other radicals, promoting aggregation and thrombogenesis [18,27,28]. 

Role of toxicant exposure in cardiovascular disease pathogenesis

**Figure 2:**
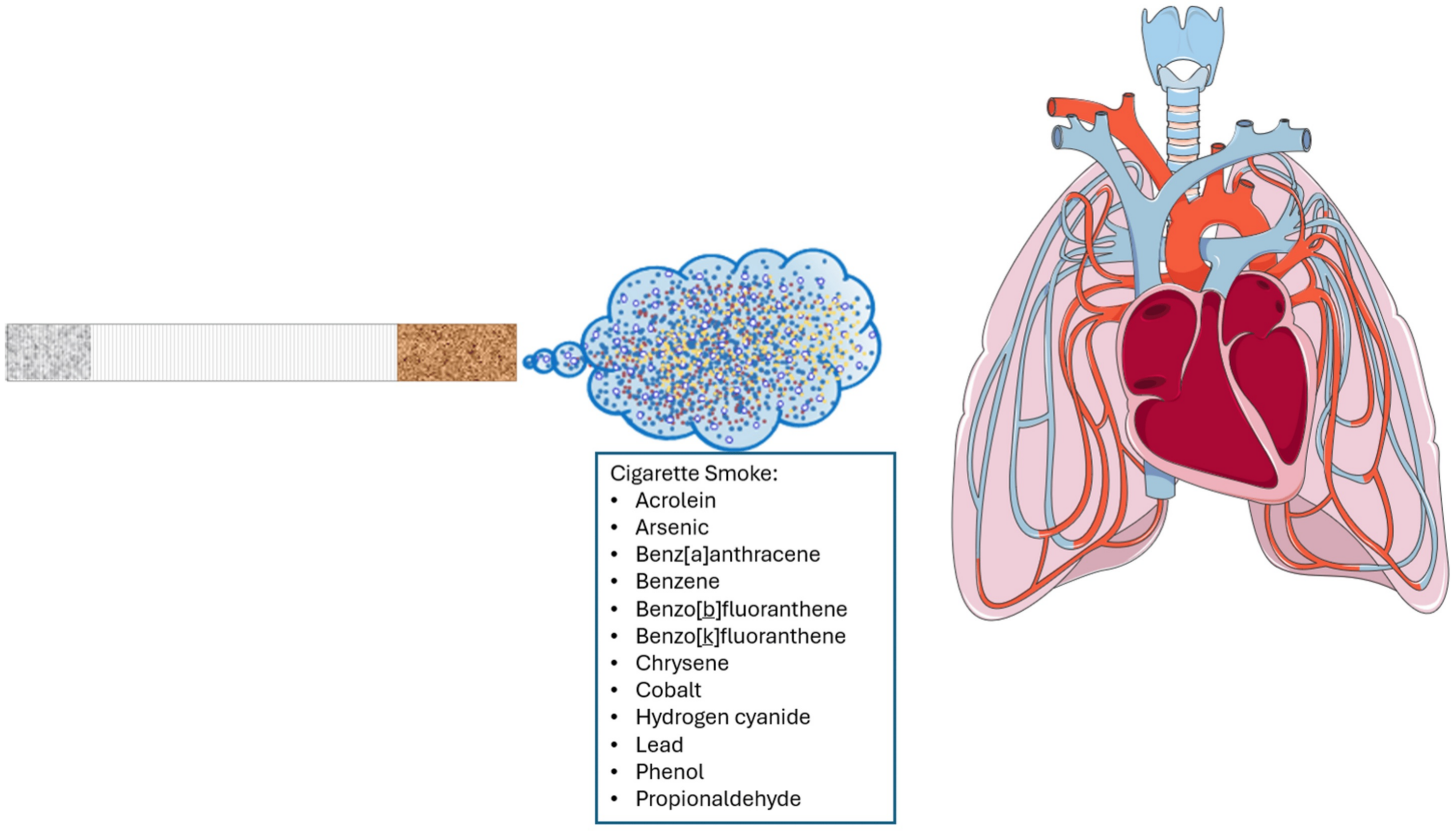
Cigarette Smoke Toxicants with Causal Link to Cardiovascular Disease. The figure shows smoke toxicants defined as cardiovascular toxicants according to the US Food and Drug Administration [31]. Figure created by Matthew Stevenson using PowerPoint (Microsoft Corporation, Redmond, USA). Constituent images (cigarette, smoke cloud, and heart and lungs) were provided by Servier Medical Art (https://smart.servier.com), licensed under CC BY 4.0 (https://creativecommons.org/licenses/by/4.0/).

The primary cause of smoking‑related disease is the long-term and repeated inhalational exposure to toxicants that are either present in the tobacco leaf and transferred into the cigarette smoke or are formed during the processes of pyrolysis and combustion in the burning cigarette [29,30]. Public health bodies state that a number of toxicants found in cigarette smoke may have a direct causal link to CVD, including acrolein, benzene, cadmium, hydrogen cyanide, lead, phenol, and propionaldehyde (Figure 2) [31,32].

The proposed mechanistic effects on the cardiovascular system of exposure to these toxicants, which contribute to CVD risk, include damage to the vascular endothelial layer and endothelial dysfunction, increased uptake of oxidised LDL by macrophages and foam cell formation, promotion of platelet aggregation and thrombosis, inflammation and oxidative stress, altered lipid metabolism, insulin resistance, and increased demand/diminished supply of myocardial oxygen [10,33,34]. Additionally, indirect effects of smoking, such as increased arterial stiffness and ensuing effects on blood pressure, may also contribute to smoking‑related CVD development [10,35]. These various impacts of cigarette smoke toxicant exposure on CVD development processes have been captured in adverse outcome pathways (AOPs) [34], which highlight the complexity of the link between smoking‑induced toxicant exposure, altered biological processes, and cardiovascular damage. 

In the past two decades, alternatives to cigarettes that deliver nicotine to users via the inhaled and oral routes but in the absence of combustion have emerged into consumer markets. The use of these products is increasing, and many studies have reported increases in electronic vaping product (EVP; also termed an e‑cigarette or electronic nicotine delivery system), heated tobacco product (HTP) and oral nicotine pouch (ONP) use globally. The use of a so‑called next‑generation tobacco and nicotine product (NGP) still involves exposure to nicotine, which, potentially along with other components of novel products such as propylene glycol and glycerine found in EVP liquids, has acute and short-lived cardiovascular effects such as increased blood pressure and heart rate [36,37]. It has been reported that these effects may potentially precipitate acute cardiovascular events among those with underlying CVD [36].

However, the use of NGPs either significantly reduces or eliminates exposure to other chemical toxicants responsible for initiating or promoting smoking‑related CVD [38-44]. Thus, these products may offer the potential to reduce CVD risk among smokers who exclusively switch to their use [38-40,45-47]. In this regard, a recent expert consensus group recommended that “Tobacco users who have been unable/unwilling to quit using current best evidence-based approaches, should switch completely to nicotine-containing e-cigarettes to reduce exposure to cardiovascular toxicants” and also that “Tobacco users who have been unable/unwilling to quit using current best evidence-based approaches, should switch completely to nicotine-containing e-cigarettes to improve measures of cardiovascular function” [48]. 

The aim of this narrative review is to assess the scientific literature regarding the health impacts of the use of NGPs, and specifically their potential impact on CVD risk among smokers who switch. This review focuses on four main elements: what has been reported from in vitro and in vivo CVD modelling studies; findings from human clinical studies among smokers who switch to using NGPs; assessments of the CVD risk impact of these products at the population level; and how regulators are assessing CVD risk and what determinations have been made. This provides important insight into the impact of NGPs on cardiovascular health.

Parts of this work were previously presented at the Global Forum on Nicotine conference on June 5th, 2026. 

## Review

In vitro and in vivo studies demonstrate the potential for NGPs to reduce cardiovascular risk

In Vitro Studies

A number of in vitro models mapping human CVD processes have been developed, which can be used to estimate the cardiovascular risk potential of both cigarettes and NGPs [49]. These models variously reflect individual pathogenic processes within the complex cascade of events that occur during atherosclerotic plaque formation and typically use cultured cells relevant to the cardiovascular system, such as endothelial and vascular smooth muscle cells. Some of these models map directly onto human disease processes. For example, exposure of cultured endothelial cells to cigarette smoke extracts in vitro elicits the expression of intercellular adhesion molecule‑1 (ICAM‑1; [50]). This marker is elevated among smokers, and its levels are decreased following smoking cessation [51,52]. Other models recreate functional pathogenic processes in vitro. One example is the endothelial migration (scratch wound) assay, which is an in vitro model for endothelial damage and repair that recreates an initiating step in CVD pathogenesis [53]. In this section, we review findings from in vitro studies that have assessed the impacts of aerosol extracts from NGPs relative to cigarette smoke extracts since these provide information most relevant to assessing the CVD risk reduction potential of these products.

Several studies have assessed the impact of EVP and HTP aerosols relative to cigarette smoke on various CVD‑related endpoints using simple cellular models of the vasculature. In these studies, in which vascular cells are cultured under static conditions, cells were exposed to extracts of aerosols generated from EVPs and HTPs and cellular responses were compared to those in cells exposed to cigarette smoke extracts. In an early study using cultured cardiac myoblasts (precursor cells to cardiac muscle cells), EVP aerosol extracts were significantly less cytotoxic than smoke extracts [54], and differential responses to EVP aerosol extract exposure were observed depending on the e‑liquid flavour and EVP power output used. In a similar study using human pluripotent stem cell-derived cardiomyocytes, the cardiotoxic potential of aerosol extracts from various EVPs and HTPs was significantly (up to 10‑times) lower than that of cigarette smoke extracts [55].

**Figure 3:**
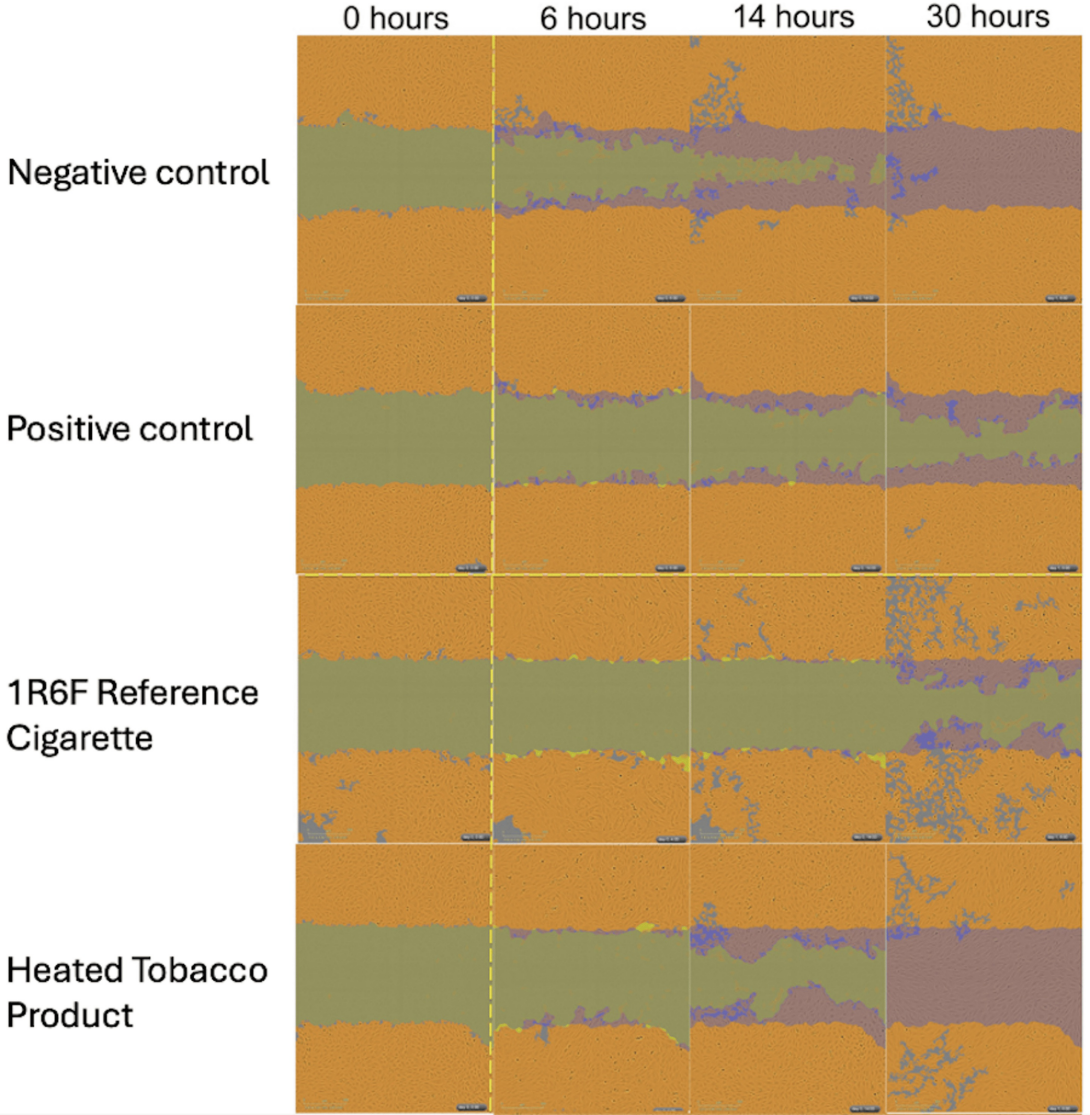
Impact of Cigarette Smoke and HTP Aerosol on Endothelial Damage Repair in a Scratch Wound Assay. Phase contrast images illustrating scratch-wounded cell cultures over the assay period (30 h). Cells were wounded, then the test articles (negative control (phosphate-buffered saline), positive control (cytochalasin D), 1R6F reference cigarette smoke extract, and heated tobacco product aerosol extract) were added at 0 h. The different colours represent the masks used to calculate relative wound healing: green = scratch area (no cells); orange = original cell-populated area following scratch wounding; purple = area of cells populating the original wound area; blue = cell-free area (following cell migration). Image reproduced with permission from Chapman et al. [57]. Abbreviation: HTP, heated tobacco product.

Though the cells used in these studies may not be entirely relevant to atherosclerotic CVD, they still remain indicative of cardiovascular cytotoxicity responses to EVP and HTP aerosols relative to cigarette smoke. Perhaps more relevant to CVD, some studies have used immortalised endothelial cell lines and assessed cellular responses to exposure. Su et al [56] reported reductions in loss of cell viability, apoptosis, adhesion molecule and inflammatory marker expression, and ROS generation, in human umbilical vein endothelial cells exposed to EVP aerosol extracts compared with those exposed to cigarette smoke extracts. In scratch assays of endothelial damage and repair using immortalised endothelial cell lines, the inhibition of endothelial cell migration and wound healing following 1R6F reference cigarette smoke exposure was not seen when cells were exposed to aerosol from tobacco‑ and menthol-flavoured HTPs (Figure 3 and [57]).

Similarly, inhibition of endothelial cell migration following exposure to aqueous extracts of 3R4F reference cigarette smoke was not seen when cells were exposed to aqueous extracts of EVP [53] and HTP [58] aerosols. In multiplex assays using many different cell lines, including immortalised endothelial cells, blood mononuclear cells and vascular smooth muscle cells and assessing various markers related to inflammation and CVD, aerosols from HTPs, EVPs and a hybrid HTP/EVP were weakly active compared to 3R4F reference cigarette smoke [59]. Overall, the use of these simple, static in vitro models demonstrates reduced propensity of novel tobacco and nicotine product aerosols to damage the cardiovascular system and contribute to CVD development compared with cigarette smoke. 

More complex, non-static models have also been utilised to assess CVD‑related effects of exposure. Flow models, which employ fluidics systems to recreate in vitro the extracellular environment and physiological stresses exerted on cells within the cardiovascular system, have been used in some studies. Makwana et al [60] reported findings from a study in which endothelial monolayers within a microfluidics system were exposed to either cigarette smoke or EVP aerosol, subsequent to which a monocyte adhesion assay was performed by perfusing the exposed endothelial layer with cultured THP‑1 cells [60]. This assay recreates in vitro the migration of immune cells into the growing atherosclerotic plaque. In this assay, the adhesion of monocytes was significantly reduced following EVP aerosol exposure compared with cigarette smoke extract exposure, an effect likely underpinned by the further finding of elevated ICAM‑1 expression in endothelial cells exposed to cigarette smoke but not in those exposed to EVP aerosol [60].

A similar study using endothelial cells cultured under flow conditions also reported reduced monocyte adhesion, oxidative stress, and expression of ICAM-1 and other chemoattractants and inflammatory mediators in cells exposed to EVP and HTP aerosol extracts compared to cells exposed to cigarette smoke extracts [61]. Similar findings, in terms of monocyte adhesion and the involvement of ICAM‑1, were reported by Ohashi et al in a study demonstrating a reduced impact of aerosol extracts generated from several different types of HTP compared with cigarette smoke [62]. That study also demonstrated reduced levels of the inflammatory marker interleukin-1β (IL-1β) from macrophages derived from THP‑1 cells exposed to HTP aerosol extracts compared with those exposed to cigarette smoke extracts [62].

Additionally, Giebe et al reported findings from a flow‑based microfluidics model of the endothelial layer [63]. This demonstrated a reduced biological impact, in terms of monocyte adhesion and the expression of various adhesion and inflammatory markers, following exposure to EVP and HTP aerosol extracts compared with exposure to cigarette smoke extracts. Importantly, the complexity of this model, in which cultured endothelial cells were cultured under conditions that mimicked those experienced by atherosclerosis‑prone vascular cells in vivo, as well as the study’s further assessment of endothelial migration in this fluidics model with a similar finding of reduced impact of EVP and HTP aerosols [63], enhances its validity and the plausibility of the findings. In an even more complex and biologically‑relevant model, Poussin et al established a 3‑dimensional model of endothelial microvessels cultured on a chip under flow conditions [64].

Similar to previous studies, HTP aerosol extract exposure elicited reduced monocyte adhesion and expression of ICAM‑1 compared with cigarette smoke exposure, as well as reduced release of the inflammatory marker tumour necrosis factor α (TNFα) and depletion of glutathione (GSH), a marker of oxidative stress [64]. These findings mimicked those previously reported by the same group in a model using endothelial cells cultured under static conditions, studies that also demonstrated reduced expression of genes encoding other inflammatory mediators, various adhesion molecules, and markers of oxidative stress, in endothelial cells exposed to HTP extracts relative to those induced by cigarette smoke extract exposure [65,66]. A similar static model using cultured VSMC instead of endothelial cells was also utilised, a model that relates to atherosclerotic plaque growth due to proliferation of these cells and other cellular processes within the growing plaque [67]. This further study demonstrated that HTP aerosol extract exposure had a lower impact on many processes relevant to CVD development and progression than cigarette smoke extract exposure [67], a finding that this and other studies suggest may be related to reduced carbonyl yields in the HTP aerosols [61,65-67].

Most recently, the use of a ‘vascular-on-a-chip’ in vitro model, which mimics the characteristic physiology of the human vasculature to assess initiating events in atherosclerosis, demonstrated that endothelial barrier impairment, monocyte adhesion and monocyte migration were significantly lower when the system was exposed to HTP aerosol compared with cigarette smoke exposure [68]. Furthermore, these pathogenic processes were not significantly different between HTP aerosol exposure and a solvent control. These findings support the reduced atherosclerosis risk potential of HTPs compared with cigarettes [68].

In Vivo Studies

The number of in vivo studies conducted to assess the potential impact of NGPs on cardiovascular health relative to cigarettes is lower than that for in vitro studies, but these studies still give an indication of differential effects of smoke compared with exposure to novel product aerosol. Phillips et al [69] reported findings from an 8‑month study in which apolipoprotein E‑deficient (Apoe-/-) mice, which, due to genetic ablation of a lipoprotein metabolising enzyme, develop hypercholesterolaemia that promotes the development of atherosclerotic plaques [70,71]. Using this model, mice were whole-body exposed to either air (sham), cigarette smoke, or HTP aerosol, 5 days a week for up to 8 months. Additional arms also examined ‘cessation’ (stopping cigarette smoke exposure) or ‘switching’ (changing from smoke exposure to HTP aerosol exposure following 2 months of smoke exposure) [69].

Cigarette smoke exposure increased the area of the aortic arch affected by plaque development and also increased the plaque volume and aortic occlusion. Exposure to HTP aerosol caused significantly lower plaque area, volume and aortic occlusion compared with cigarette smoke exposure, to a degree that was not significantly different from the area in mice in the ‘cessation’ and ‘switching’ arms of the study [69]. Using the same model, the authors further demonstrated that during a shorter, 6-month exposure period with 3‑month ‘switching’ and ‘cessation’ arms, plaque area and volume were significantly lower in mice exposed to HTP aerosol compared with cigarette smoke. Again, these parameters approximated to those seen with ‘cessation’ or ‘switching’. These reductions in CVD‑related endpoints were associated with reduced toxicant exposure and reduced haematological parameters [72], reductions in impacts on left ventricular structure, systolic ejection fraction and fractional shortening, and reductions in cardiovascular transcriptome modification [73].

A similar study assessing the impacts of EVP aerosol exposure also showed significantly reduced plaque surface area compared with cigarette smoke exposure, and as previously, the amount of the aorta affected by plaque formation was not significantly different in the EVP aerosol-exposed mice compared with sham (air) exposure [74]. This reduced impact was associated with reduced toxicant exposure, reduced levels of biomarkers of oxidative stress and inflammation, improved haematological and cardiac function parameters, and lower perturbations of the cardiac transcriptome and proteome [75]. In the most recent assessment of the impact on CVD development in Apoe-/- mice, total atherosclerotic lesion area was higher in animals exposed to cigarette smoke than in those exposed to EVP aerosol [76]. This effect was greater in female mice, and also among those animals fed a Western (high fat and calorific content) diet, which may suggest a complex interaction between exposure type, gender and other CVD risk factors such as diet, on the development of atherosclerosis [76].

Using a different mouse model (C57BL/6 mice, which are genetically predisposed to develop obesity, impaired glucose tolerance and atherosclerosis when fed a high-fat diet) [77], similar findings related to potentially reduced atherosclerotic propensity have been reported. In this model, Olfert et al [78] demonstrated that following an 8‑month exposure to either cigarette smoke or EVP aerosol, vascular stiffness was lower in the EVP aerosol‑exposed animals, vascular responses to the vasoconstrictor phenylephrine were improved, and improvements were also seen in cardiac ejection fraction and fractional shortening [78]. Other parameters related to CVD development were, however, not different between the EVP aerosol‑exposed and smoke‑exposed mice. While differences were apparent between animals exposed to EVP aerosols and those exposed to air, this may be due to the use of intense puffing on the EVP when creating EVP aerosols, as well as an intense exposure regimen, which may not necessarily reflect human use conditions. In another mouse model using A/J mice, although primarily conducted to assess chronic toxicity and carcinogenicity, endpoints related to CVD development and haematological parameters were improved in mice exposed to HTP aerosol compared to those exposed to cigarette smoke [79]. 

Cardiovascular adverse outcome pathway studies

Adverse outcome pathways are frameworks that facilitate the collection of mechanistic information derived from toxicological science in a structured manner to assist in establishing causal relationships between molecular and cellular events that lead from perturbation of the biology to adverse health effects [80]. An AOP for cigarette smoke‑mediated oxidative stress in plaque formation has recently been developed [34]. This AOP both highlights the complexity of the impact of cigarette smoke on the cardiovascular system at the cellular, tissue and organ levels, and presents an opportunity to develop preclinical assays and biomarkers with which to improve upon our ability to assess the relative CVD risk of NGPs [34]. A recent paper has used a cardiovascular AOP framework to examine the cardiovascular impacts of cigarette smoking and EVP use, finding that EVP use presents a lower CVD risk than cigarette smoking [81]. While the use of this AOP framework did suggest some potential for adverse CVD outcomes related to EVP use, the publication by Ding et al [81] did not appear to take into account the likely degree of exposure based on the levels of toxicants in EVP aerosols, a fundamental principle of toxicological risk assessments.

Furthermore, data from in vitro and in vivo studies utilised by Ding et al [81] may be confounded by the generation of EVP aerosols in a manner that does not replicate human use conditions. Overall, AOPs provide an opportunity to better understand the mechanisms associated with cigarette smoking‑induced CVD and any reductions in risk associated with the use of NGPs, but in doing so, they must rigorously adhere to the scientific principles of toxicological risk assessment, including determining the degree of exposure, and ensure that any CVD‑related effects discovered are based on testing conditions that approximate to human use. 

Overall, the findings from in vitro and in vivo studies demonstrate the potential for NGPs to reduce CVD risk. While some of these studies could be improved upon by increasing the length of cigarette smoke exposure before ‘cessation’ or ‘switching’, or by generating aerosols and exposing cells in vitro or animals in vivo under scenarios that better approximate to human use, the convergence of findings from the use of different approaches strongly supports a reduced CVD risk potential of NGPs. 

Clinical studies demonstrate the potential for reduced cardiovascular risk

Studies Assessing Biomarkers of Exposure for Toxicants Linked to Cardiovascular Disease

**Figure 4:**
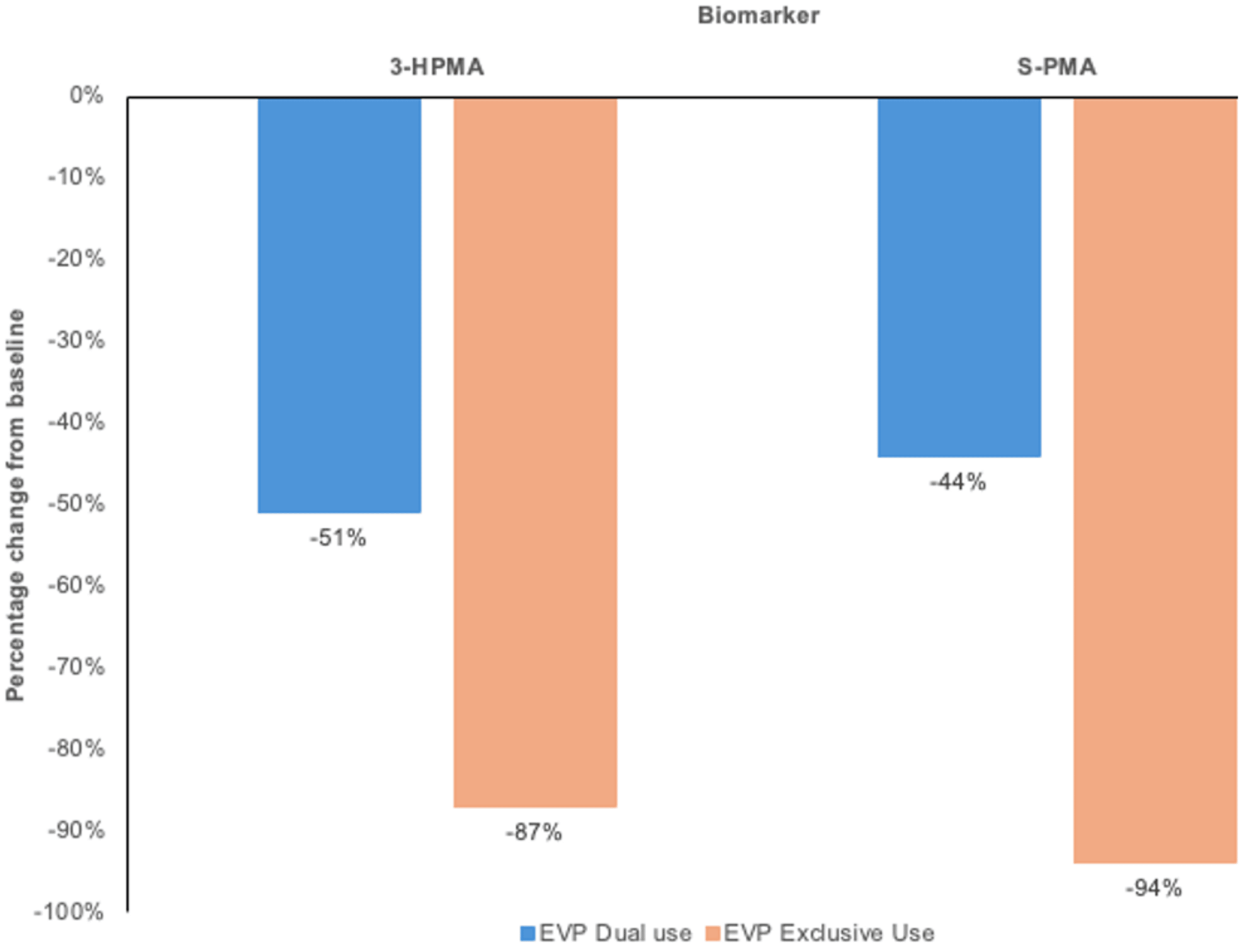
Changes in Biomarkers of Exposure to Cardiovascular Toxicants Among Smokers Switching to Using an EVP. Orange bars represent participants who exclusively used EVPs ad libitum from Day 10 of the Study (n = 14). Blue bars represent participants reporting dual use (50% usual brand cigarette smoking and EVP use ad libitum from Day 10 of the Study; n = 12). Values are expressed as a percentage change from baseline levels (cigarette smoking) measured on Day 1, with the percentage reduction also shown below each bar. Data adapted from Morris et al [97]. Abbreviations: 3‑HPMA, 3-hydroxypropylmercapturic acid (biomarker for acrolein exposure); EVP, electronic vaping product; S-PMA, S-phenylmercapturic acid (biomarker for benzene exposure).

A number of longitudinal clinical studies have assessed the impact of switching from cigarette smoking to using NGPs on biomarkers of exposure (BoE) to smoke toxicants, some of which have a defined link to CVD risk [31]. Many studies have assessed changes in BoE among smokers switching to the use of EVPs [82-100], and an example of the reductions in BoE to cardiovascular toxicants upon switching to EVP use is presented in Figure 4.

Other studies have assessed changes in BoE upon switching to the use of HTPs (both electrically heated and heated using a lit carbon heat source [96,101-130], ONPs [131], and other types of oral tobacco products [132,133]. While the various studies assessing EVPs, HTPs, ONPs and other oral tobacco/nicotine products did not capture changes in cardiovascular risk per se, they all indicate that BoE to chemical toxicants with a known link to CVD, such as carbon monoxide (CO), acrolein, benzene and hydrogen cyanide [31], are significantly reduced or eradicated among switching smokers. These biomarkers can serve as intermediate endpoints for the health consequences associated with tobacco and nicotine product use [99], and in many instances, biomarker reductions approached, or were similar to, those seen with complete abstinence from cigarette smoking or the use of any tobacco and nicotine products [82,83,87,89,90,92-96,99,100,104-108,111-115,118,119,121,131-134].

The reductions reported are also in line with the findings from analytical chemistry analyses, which show that many toxicants found in cigarette smoke are either reduced in or absent from EVP and HTP aerosols and ONP extracts [38,40,46] [44]. Notably, other toxicants with a link to CVD, such as benz[a]anthracene, benzo[b]fluoranthene, benzo[k]fluoranthrene, and phenol, were not measured in these clinical studies due to the lack of appropriate and validated methods for their measurement in biological samples. These toxicants are, however, found at significantly lower levels, and in many cases are below detectable levels, in aerosols or extracts from NGPs compared with cigarette smoke (e.g., [135-138]. As such, the BoE studies may underestimate the CVD risk reduction potential of novel tobacco and nicotine product use compared with cigarette smoking. 

Findings from cross‑sectional studies assessing BoE among those who smoke or use NGPs have also been reported [139-146], which are perhaps more indicative of real‑world exposure than studies conducted either in a clinical confinement setting or during enforced ambulatory product use. These studies have similarly found that compared to smokers, those who exclusively use HTPs [139], EVPs [140-145] or ONPs [146] have significantly lower levels of BoE for toxicants linked to CVD risk. In the studies in which comparisons were made, BoE levels were similar among exclusive EVP and ONP users compared to those seen among former smokers who were not using other tobacco and nicotine products [139,140,142,143,146] or never‑smokers [142-144,146]. This suggests that complete switching has the same benefit, in terms of reducing exposure to CVD risk‑related toxicants, as both complete abstinence and never‑smoking. 

Studies assessing biomarkers of potential harm

**Figure 5:**
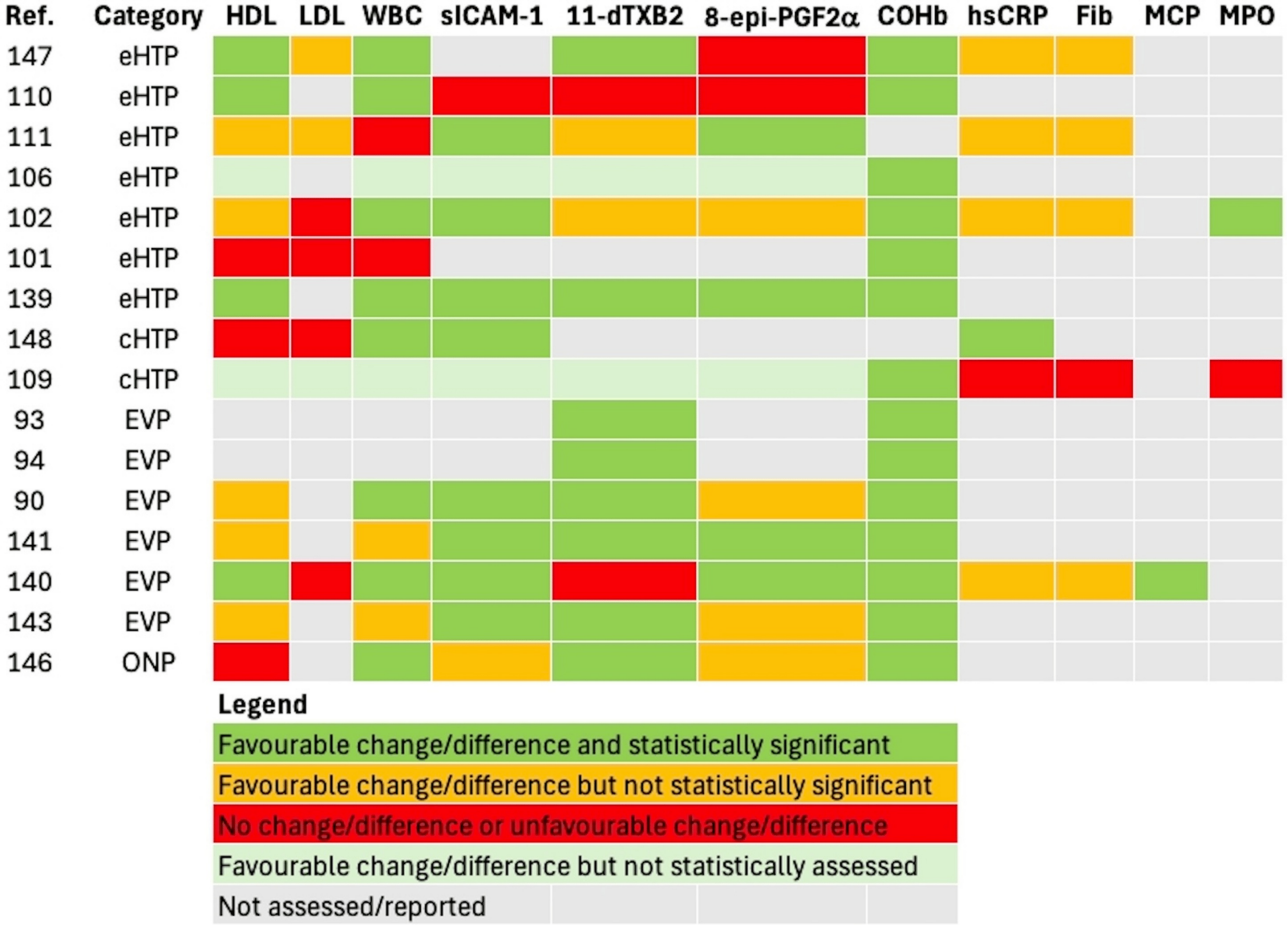
Summary of Changes/Differences in Cardiovascular Disease Related BoPH from Longitudinal and Cross Sectional Studies Assessing NGPs. Note: details for the cited references used in assimilating BoPH data can be found in Table 1. Image credits: Ian M. Fearon. Abbreviations: BoPH, biomarker(s) of potential harm; cHTP, carbon-heated tobacco product; COHb, carboxyhaemoglobin; 11‑dTXB2, 11‑dehydrothromboxane B2; eHTP, electronically-heated tobacco product; 8‑epi‑PGF2α, 8‑epi‑prostaglandin F2α; EVP, electronic vaping product; Fib, fibrinogen; HDL, high density lipoprotein cholesterol; hsCRP, high‑sensitivity C-reactive protein; LDL, low density lipoprotein cholesterol; MCP, monocyte chemoattractant protein; MPO, myeloperoxidase; NGP, next‑generation tobacco and nicotine product; ONP, oral nicotine pouch; ref, reference publication; sICAM‑1, soluble intercellular adhesion molecule‑1; WBC, white blood cell.

Longitudinal switching studies and cross‑sectional studies have also assessed potential changes in CVD risk among smokers switching to using NGPs, using biomarkers of potential harm (BoPH) or biomarkers of biological effect (BoBE) linked to CVD risk or associated with inflammation and oxidative stress. Findings from these studies, in terms of changes or differences in BoPH related to CVD risk upon switching to EVP, HTP or ONP use, are summarised in Figure 5, and the details of the studies providing BoPH data can be found in Table 1. 

**Table 1:** Publication Details for BoPH Studies Assessing NGPs Presented in Figure 5. Abbreviations: BoPH, biomarker(s) of potential harm; cHTP, carbon-heated tobacco product; COHb, carboxyhaemoglobin; eCO, exhaled carbon monoxide; eHTP, electronically-heated tobacco product; EVP, electronic vaping product; NGP, next‑generation tobacco and nicotine product; ONP, oral nicotine pouch.

Publication	Category	Duration	Comments
Roethig et al, 2008 [147]	eHTP	12 months	
Ludicke et al, 2019 [110]	eHTP	6 months	
Haziza et al, 2020 [111]	eHTP	3 months	
Gale et al, 2022 [106]	eHTP	12 months	eCO assessed instead of COHb
Ansari et al, 2024 [102]	eHTP	12 months	
Chu et al, 2025 [101]	eHTP	21 days	eCO assessed instead of COHb
Ansari et al, 2025 [139]	eHTP	Cross-sectional (>2 years)	
Ogden et al, 2015 [148]	cHTP	24 weeks	
Bosilkovska et al, 2020 [109]	cHTP	90 days	
Kanobe et al, 2022 [93]	EVP	7 days	
Kanobe et al, 2023 [94]	EVP	7 days	
Edmiston et al, 2022 [90]	EVP	24 weeks	
Oliveri et al, 2020 [141]	EVP	Cross-sectional (>6 months)	Data for users of any type of EVP
Shiffman et al, 2024 [140]	EVP	Cross-sectional (>6 months)	
Haswell et al, 2023 [143]	EVP	Cross-sectional (>6 months)	Data from per protocol population
Azzopardi et al, 2023 [146]	ONP	Cross-sectional (>6 months)	Data from per protocol population

In an evaluation of the impact of switching to exclusive HTP use for 12 months, Roethig et al [147] reported pathophysiologically‑favourable changes in several cardiovascular risk markers including WBC counts, haemoglobin, haematocrit, 11‑dehydrothromboxane B2 (11‑dTXB2) and high‑density lipoprotein (HDL) cholesterol. This finding has been replicated more recently, in studies assessing switching impacts of more modern HTPs that have reported favourable changes in 8‑epi‑prostaglandin F2α (8‑epi‑PGF2α), 11-dTXB2, soluble intracellular adhesion molecule-1 (sICAM‑1), HDL cholesterol and WBC counts following 3, 6 or 12 months of switching [106,110,111]. In the most recently published assessment of switching to an electrically heated HTP, at both 6 and 12 months, favourable changes in several CVD risk‑related BoPH were reported, including sICAM‑1, 11‑dTXB2, fibrinogen, WBC counts, 8‑epi‑PGF2α and high sensitivity C-reactive protein (hsCRP) [102], a study that also provided some evidence of a benefit of substantially reducing cigarette consumption.

While other studies have reported no impact of switching to HTP use on BoBE [101], even despite detecting changes in BoE, this is likely due to the short time period (21 days) of the study, which is insufficient for biomarker changes to occur [149]. However, blood transcriptomic responses have been reported over a short time period (5 days) among smokers who switch to using an HTP [117]. These findings from switching studies of potential CVD risk reductions among switching smokers are supported by a cross‑sectional study assessing differences between continuing smokers and those who have switched to HTP use for a period of two or more years [139]. For all CVD‑related endpoints, which included WBC counts, 8‑epi‑PGF2α, HDL cholesterol, central vascular augmentation index (central AIx, a measure of vascular stiffness), sICAM‑1 and 11‑dTXB2, values for HTP users were significantly different, in a favourable direction, from those among continuing smokers [139]. 

Similar to the studies on electrically‑heated HTPs, when assessing the impact of switching to exclusive use of carbon‑heated HTPs for periods from 12 weeks to 12 months, haematocrit, platelet counts, sICAM‑1 and WBC counts were significantly lower among those who switched to using an HTP compared to baseline or to continued smoking [109,148]. 

For EVPs, switching studies have also found favourable changes in BoPH/BoBE. Even over a short time period of 5 to 7 days, Kanobe et al [94] and [93] reported significant reductions in 2,3‑dinor thromboxane B2 (2,3‑dinor TXB2) and 11‑dTXB2, markers of platelet activation, among smokers who exclusively switched to using one of three types of pod‑system EVPs. Over a similar period of time, D’Ruiz et al [150] reported reductions in systolic and diastolic blood pressures and heart rate, following both complete and partial switching to EVP use. In a longer‑term assessment of switching, Edmiston et al [90] reported significant and/or favourable changes in 11‑dTXB2, WBC counts, sICAM‑1 levels, HDL cholesterol and 8‑epi‑PGF2α among smokers who switched to using a pod‑based EVP for 24 weeks. These findings for EVP, like those for HTPs, are supported by real-world, cross‑sectional observational studies. Oliveri et al [141] found that among those who had been using EVPs for six months or more following 10 or more years of cigarette smoking, WBC counts and the levels of 11‑dTXB2, 8‑epi‑PGF2α and sICAM‑1 were lower among the EVP users compared with continuing smokers. Some differences are also apparent between users of different types (pod or tank system) of EVPs [141].

Similarly, Shiffman et al [140] reported that WBC counts, monocyte chemoattractant protein‑1 (MCP‑1) and haemoglobin A1c (HbA1c) were significantly lower, and HDL cholesterol was significantly higher, among former smokers who had switched to using the JUUL pod-based EVP (Juul Labs, Inc., San Francisco, USA) for at least 6 months compared with continuing smokers [140]. Haswell et al [143] also found significantly lower levels of the BoPH 11‑dTXB2 and sICAM‑1 among those who had been using EVPs for at least six months compared with smokers, although in that study 8‑epi‑PGF2α, WBC counts and HDL cholesterol levels were not significantly different between those two groups, nor were other more functional markers of cardiovascular health [143]. Potentially, more prolonged switching is required to see improvements in other biomarkers and functional endpoints. Finally, a cross‑sectional study has also found that, among ONP users, BoPH related to CVD risk, including sICAM‑1, 8‑epi‑PGF2α, 11‑dTXB2 and WBC counts, were lower among those who had been using Velo ONPs (British American Tobacco p.l.c., London, UK) for at least six months compared with cigarette smokers [146]. This effect was only significant, however, for WBC counts and 11‑dTXB2, and it is also notable that the levels of these two BoPH were similar among the ONP users to those seen among former smokers and never smokers [146].

Also, and similar to the cross‑sectional study of EVP users [143], other BoPH related to CVD risk and functional markers of CVD health were not significantly different between the ONP users and cigarette smokers [146]. This again suggests that a more prolonged period of switching is necessary to realise the full potential benefit on cardiovascular health and CVD risk.

Several other studies have assessed the relative impact of acute EVP use compared with cigarette smoking on cardiovascular endpoints, and to a degree, these studies mirror those impacts reported in switching studies. In cohorts of smokers and non‑smokers, Carnevale et al [151] assessed markers of oxidative stress, nitric oxide bioavailability, and flow‑mediated dilation (FMD; a marker of vascular responsiveness) when subjects smoked a cigarette or used EVPs. While both cigarette smoking and EVP use increased the levels of the ROS‑producing soluble nicotinamide adenine dinucleotide phosphate (NADPH) oxidase‑derived peptide (sNOX2dp), a marker of oxidative stress and activation of platelets and immune cells, as well as 8‑Isoprostaglandin F2α (8‑iso‑PGF2α), and decreased nitric oxide bioavailability, these impacts were significantly lower following EVP use compared with cigarette smoking. The impact on FMD, however, was not different between cigarettes and EVPs [151], an effect that may be due to a short-term effect of nicotine rather than something attributable to toxicant exposure.

In another study assessing the impact of EVP and HTP use on oxidative stress, antioxidant reserve, platelet activation, FMD and blood pressure endpoints in smokers [152], similar to Carnevale et al [151], acute negative impacts of the use of EVPs and HTPs were observed. These included effects on sNOX2dp levels, hydrogen peroxide production, soluble CD40 ligand levels (a protein shed by platelets and lymphocytes that plays a role in inflammation and immune responses), P‑selectin levels (an adhesion molecule that plays a role in WBC adherence to the endothelium), FMD and blood pressure [152]. However, also similar to Carnevale et al [151], these effects were lower, significantly in many cases, for EVP and HTP use compared with cigarette smoking [152]. In a further study assessing slightly longer-term cardiovascular impacts of EVP use among smokers [153], within one month of switching from smoking to EVP use, significant improvements in endothelial function (assessed by FMD) were observed. Vascular stiffness (assessed using pulse wave velocity) did not improve in the overall study cohort in this time frame. However, when subjects were grouped according to their smoking history (pack‑years), those who smoked ≤20 pack-years had a significantly improved pulse wave velocity. Notably also, several other secondary markers, including AIx, LDL, and hsCRP, were not significantly different from baseline following one month of switching to EVP use [153].

The impact of HTP use on blood parameters and cardiovascular risk was assessed in a recent Polish case-control study comparing a group of regular HTP users with a control group of non-users [154]. Mean RBC counts were lower among HTP users; mean corpuscular volume, mean corpuscular haemoglobin, and fibrinogen levels were higher among HTP users; and the adhesion molecules vascular cell adhesion molecule‑1 (VCAM‑1) and ICAM‑1 were not different between the two groups [154]. While these findings may be suggestive of increased CVD risk among HTP users, it is notable that a history of cigarette smoking was not considered in the HTP user cohort, and the majority of subjects in that cohort (86.3%) were ever‑smokers. 

One further study assessed the relative impact of smoking and EVP use using an ex vivo atherogenesis (monocyte transendothelial migration and foam cell formation) assay among people living with human immunodeficiency virus (PLWH) [155]. Monocyte transendothelial migration and foam cell formation following smoking were increased compared with a control intervention, but following EVP use, neither were significantly different compared to the control [155]. These findings of the potential for smaller proatherogenic effects and reduced cardiovascular harm associated with EVP use relative to cigarette smoking complement previous findings from the same group of a reduced impact on arrhythmogenic heart rate variability (HRV) among PLWH when using EVPs compared with smoking [156]. Furthermore, among healthy subjects, no impact on baseline heart rate, blood pressure and HRV was found between EVP users and non‑users, although acute EVP use had an impact on arrhythmogenic HRV parameters [157], an effect that appears related to nicotine exposure.

Overall, the outcomes from these studies, along with other studies described previously, are clear. The use of EVPs may have a cardiovascular impact, but this is demonstrably less than that of cigarette smoking. Studies suggest that switching to EVP use can reverse the cardiovascular impact of smoking, even in the short term, though longer‑term and exclusive switching are required to have the greatest impact. Such a conclusion is not universal, however [158]. Underpinning the differential effects reported in some studies compared with others may be the complexity of the association between cigarette smoking and CVD development, as well as the association between smoking history and length/exclusivity of switching to EVP use on risk reversal. Future studies need to take into account these and other confounding factors in order to determine the overall impact of EVP use relative to cigarette smoking on cardiovascular function and health [158,159], taking into account the complexity of the various risk factors and pathological processes underpinning atherosclerotic CVD development and its sequelae.

Integrating findings from studies using different measures of risk, including BoE, BoPH, and functional and proteomic measures, in individuals using NGPs is required to predict future cardiovascular risk [160]. Importantly, such studies must assess the cardiovascular impacts of novel product use relative to those associated with cigarette smoking, in order that the risk reduction potential of novel products, if any, can be fully ascertained.

Population-level studies confirm the reduced cardiovascular risk associated with exclusive NGP use

The impact of NGPs on CVD at the population level has been assessed in numerous studies, mainly using data obtained from federally‑funded United States (US) national surveys such as the Population Assessment on Tobacco and Health (PATH) study [161]. These studies have focused mainly on the health impacts of EVP use, primarily because of the prevalence of EVP use in the US and since other products such as HTPs and ONPs are either not commercially available or have not yet been available for a sufficient length of time to enable the detection of population‑level effects. It should also be noted that while these studies may provide indications as to the health impact of novel products, these studies are primarily cross‑sectional in nature and therefore can only assess associations and not causation and may equally be susceptible to reverse‑causation [162-164]. Even some studies that have used a longitudinal assessment approach may also be hampered by this inability to properly assess causation. These studies may also be affected by not fully addressing confounding factors such as former smoking in the statistical models, and many have also failed to properly address the issue of the temporality of disease diagnosis relative to the initiation of product use [165]. These hindrances to assessing CVD risk associated with novel tobacco and nicotine product use may be compounded by their inclusion in systematic reviews and meta-analyses. Therefore, this section focuses on individual articles assessing CVD risk among users of NGPs. 

Early analyses of PATH data reported an association of daily EVP use with an increased risk of MI [166]. However, that paper was retracted based on the failure to relate the time of the MI to the initiation of EVP use [167], rendering the conclusion unreliable. Subsequent re-analyses of the same data showed that at least 11 of the 38 EVP users who reported having an MI suffered this event prior to initiating EVP use [168]. When these users were removed from the dataset, the adjusted odds ratio for MI among daily EVP users was found to be 0.69 [168]. If we assume that a large proportion of EVP users have a history of cigarette smoking [159,169], this finding is consistent with the hypothesis that switching to EVP use reduces CVD risk. This also concurs with findings from longitudinal and cross‑sectional clinical studies described above of reductions in biomarkers associated with CVD risk among smokers who switched to EVP use.

Hypertension, a risk factor for CVD, has also been assessed using data from Wave 3 of the US Population Assessment of Tobacco and Health (PATH) study [169]. After adjusting for potential confounders, current EVP use and current smoking were both associated with higher odds of hypertension. Notably, however, the weighted proportions of current exclusive smokers (22.4%) and former smokers (20.9%) who self-reported hypertension were significantly higher than those for current exclusive EVP users who had never smoked (7.6%) and similar to those among current exclusive EVP users who were former smokers (22.5%) [169]. This finding perhaps indicates that EVP use per se is not a cause of hypertension, and also that hypertension may persist among smokers who switch to EVP use. 

Data from the US PATH study have also been used to assess BoPH among EVP users, to test the hypothesis that the lower levels of BoE among former smokers who currently use EVPs compared with current smokers translate into a CVD risk reduction [170]. In this study, which rigorously controlled for smoking intensity and duration in the statistical models, levels of several biomarkers of CVD risk (interleukin‑6; IL-6), hsCRP, fibrinogen, sICAM‑1 and F2‑isoprostane) were not significantly different between EVP users and either former smokers/never‑users of tobacco and nicotine products (adjusted odds ratios (aORs) between 0.98 and 1,15, with the 95% confidence intervals (CIs) not spanning 1 [170]). Additionally, in this rigorous analysis, dual use of EVPs and cigarettes was associated with BoPH levels that were not significantly different to those among exclusive smokers for each of four of the five biomarkers assessed (aORs 0.97 to 1.03; 95% CIs 0.89 to 1.19), the exception being F2‑isoprostane, which was significantly higher in the dual use group (aOR 1.09; 95% CIs 1.03 to 1.15) [170]. Overall, these findings suggest that switching from smoking to exclusive EVP use is associated with CVD risk biomarker reductions, but that incomplete switching and dual use maintain the CVD risk.

In a similar study assessing BoPH using data from Wave 1 of the US PATH study [171], exclusive use of EVPs was associated with reduced levels of hsCRP (though this effect was not significant in a multivariable‑adjusted model), IL-6, sICAM‑1, fibrinogen and 8‑isoprostane. Furthermore, these biomarker levels were not significantly different between exclusive EVP users and nonusers (i.e., those who had never smoked or used EVPs) when assessed in multivariable‑adjusted models [171]. When taking into account the fact that a large proportion of exclusive EVP users have a history of cigarette smoking [159,169], this suggests CVD risk reversal among those smokers who switch to exclusive EVP use. Dual use of EVPs and cigarettes was, however, associated with similar biomarker levels as exclusive smoking, suggesting that switching to exclusive EVP use is more beneficial than partial switching. A further study by the same group reported findings that suggest these differences in biomarker levels translate into reduced CVD risk.

In their longitudinal analysis of US PATH study data between 2013 and 2019, Berlowitz et al [172] reported hazard ratios (HRs) forany cardiovascular disease of 0.66 (95% CIs 0.46 to 0.94) among exclusive EVP users compared with exclusive smokers, and 0.61 (95% CI 0.34 to 1.09) for aggregated MI, heart failure or stroke incidents [172]. Furthermore, HRs were not significant for exclusive EVP users compared with tobacco and nicotine non‑users. Similar to the biomarker findings, dual use of EVPs and cigarettes was associated with a similar CVD risk as exclusive smoking. Also analysing US PATH study data from 2013 to 2019, Mahoney et al [173] compared CVD incidence in smokers, those who transitioned to EVP use, or those who quit tobacco use, with never-users of tobacco and nicotine products. Overall, aORs for CVD incidence were 1.44 (95% CI 0.87 to 2.39) among exclusive smokers, 1.85 (95% CI 0.78 to 4.37) among dual‑users, and 1.18 (95% CI 0.33 to 4.26) among former smokers. No observations of CVD incidence were found among smokers who switched to exclusive EVP use [173], although the number of such subjects in the survey population was small (53). 

In a longitudinal analysis, prospective associations between exclusive EVP use, dual use, and smoking were assessed by Hirschtick et al [174] using data from Waves 1 to 5 (2013 to 2019) of the US PATH study. Compared to no smoking or EVP use, exclusive smoking increased the risk of MI (adjusted hazard ratio (aHR) 1.99; 95% CIs 1.40 to 2.84) and stroke (aHR 2.26; 95% CIs 1.51 to 3.39), while exclusive EVP use (MI: aHR 0.61; 95% CIs 0.12 to 3.04; stroke: aHR 1.74; 95% CIs 0.55 to 5.49) and dual use (MI: aHR 1.84; 95% CIs 0.64 to 5.30; stroke: aHR 1.12; 95% CIs 0.33 to 3.79) were not significantly associated with either outcome. Importantly, the analytical model used in this study rigorously controlled for both current and former smoking status and smoking intensity and duration (pack‑years) [174]. The wide CIs are notable, however, which were potentially due to the small number of exclusive EVP users in the study. 

Analyses of other US datasets have also assessed associations of CVD with EVP use. Analysing cross‑sectional data from the Center for Disease Control and Prevention’s 2016 Behavioral Risk Factor Surveillance System (BRFSS), Bricknell et al [175] reported covariate‑adjusted odds ratios of having suffered a stroke of 2.1 (95% CIs 1.9 to 2.4) for current every‑day smokers and 1.3 (95% CIs 1.2 to 1.4) for former smokers. While the data were not analysed in a model using current smoking as a reference point to assess stroke risk among EVP users/former smokers, the aORs for stroke association among those currently using EVPs every day and on some days were 1.62 and 1.28, respectively [175]. Further analyses of pooled 2016 and 2017 BRFSS data found that the likelihoods of CVD or premature CVD among never-smoking EVP users were no higher than those of never smokers who had also never used EVPs (aOR 1.04; 95% CIs 0.63 to 1.72 for CVD; aOR 1.01; 95% CIs 0.56 to 1.83 for premature CVD). This analysis also found a significantly higher CVD risk among dual‑users of cigarettes and EVPs, both for daily and occasional EVP use [176]. However, the lack of adjustment in the statistical models for the intensity of smoking potentially renders such a conclusion unreliable.

Further analyses of the same BRFSS 2016‑2017 dataset demonstrated that stroke risk was no different between exclusive EVP users and nonsmokers (aOR 0.69; 95% CIs 0.34 to 1.42), and further that the odds of stroke were lower for exclusive EVP users than exclusive smokers (aOR 0.43; 95% CIs 0.20 to 0.93) [177]. The latter finding, considering that the majority of EVP users are former smokers, can be interpreted as a reduction in stroke risk associated with exclusive switching.

In an analysis of more recent BRFSS data from 2020, the association between EVP use, sleep duration, and CVD risk was assessed [178]. Unlike current smokers, who had a statistically significantly higher likelihood of CVD compared with never smokers, current EVP users were no more likely (aOR 1.170; 95% CIs 0.969 to 1.412) to suffer from CVD compared with never users [178]. This is indicative of a lower CVD risk of EVP use compared with cigarette smoking. Furthermore, CVD risk among former smokers/EVP users was not statistically significantly different from that among never users of either product (aOR 1.106; 95% CIs 0.833 to 1.470). In the small number of never‑smoking EVP users in the study, current EVP use was not associated with significantly increased CVD risk, which also emphasises the lack of CVD risk of EVP use compared with cigarette smoking. Dual use of cigarettes and EVP use was, however, associated with significantly increased CVD risk (aOR 1.788; 95% CIs 1.367 to 2.339) [178]. 

Using data from the US National Health eHeart Study, a cardiovascular cohort study, exclusive EVP use, compared to no product use, was associated with lower general health scores and with responding ‘yes’ to having chest pain, palpitations, coronary heart disease and arrhythmia [179]. However, an important omission in the analyses was the lack of adjustment for former smoking in the EVP user cohort. From analysing another US national dataset, obtained in the National Health and Nutrition Examination Survey (NHANES) from 2015 to 2018, Patel et al [180] assessed the relationship between EVP use and stroke. Although the prevalence of stroke was significantly lower among EVP users (1.57%) and dual users (20.39%) compared with smokers (78.04%), multivariable regression analysis reported a significantly higher risk of a history of stroke (aOR 1.15; 95% CIs 1.15 to 1.16) among EVP users compared with smokers [180]. However, the history of smoking did not appear as a confounder that was controlled for in the regression model, and it is also unclear as to the temporality of the association, such that EVP use could have been initiated after the stroke occurrence. Furthermore, this paper has recently been retracted due to ‘several major errors in the data analysis, raising concerns about the validity of the findings’ [181]. Most notably and potentially underpinning the retraction, the article claimed to have analysed data from 266,058 NHANES survey respondents, a number far greater than the number of respondents in that survey.

Using pooled data from the 2016 and 2017 US National Health Interview Survey (NHIS), Farsalinos et al [182] assessed whether EVP use was associated with coronary heart disease and MI. No statistically significant association between EVP use and CVD was found, and inconsistent associations were observed from the analyses of each year separately. In contrast, strong and consistent control associations were observed for all established risk factors for CVD, including hypertension, hypercholesterolaemia, diabetes, and cigarette smoking [182]. Further analyses of US National Health Interview Survey data from 2014 to 2019 found that EVP use was associated with having had an MI, but this association varied significantly when controlling for smoking history among EVP users [162]. Among those without a history of smoking, the use of EVPs, either past or present, was not associated with lifetime MI incidence. Furthermore, when a number of demographic and clinical variables were controlled, EVP use was associated with lifetime MI incidence only among current smokers [162]. 

In other analyses of NHIS data, this time between 2014 and 2018, exclusive EVP users had higher odds (aOR 1.244; 95% CIs 1.048 to 1.477) compared with nonusers/nonsmokers for the development of hypertension, but did not have an elevated risk of stroke, diabetes mellitus, coronary artery disease, or MI [183]. Notably also, dual use of EVPs and cigarettes was associated with higher odds of hypertension, stroke, diabetes mellitus, coronary artery disease, and MI, suggesting that exclusive switching is necessary to confer a benefit in terms of reducing CVD risk. Using the same National Health Interview Survey dataset but over an extended period between 2014 and 2021, Alzahrani et al [184] reported a significant association between EVP use and MI (aOR 2.62; 95% CIs 1.44 to 4.77) among never-smokers.

However, subsequent reanalysis of the same dataset by Plurphanswat et al [185], but with a broader and more appropriate control of confounding variables, found only 12 heart attacks among EVP‑using never‑smokers, which is well below the number of cases required for stable logistic regression estimates. Of these 12 subjects, the majority were aged 50 or older, half had high cholesterol and coronary heart disease, three-quarters were diabetic, and all were obese [185]. In two of the years from which data were analysed, no heart attack cases were reported. Using a stepwise approach to systematically investigate the contribution of confounding variables to heart attack risk, omitting the age variable caused the OR to fall from 2.48 (95% CIs 1.35 to 4.45) to 0.80 (95% CIs 1.45 to 1.43). This demonstrates that the association between heart attack and EVP use is largely moderated by age. Further, similar analyses also showed no association of diagnosis with coronary heart disease or stroke with EVP use [185]. 

In the most recent analysis of US PATH study data, Behrooz et al [186] explored the associations between EVP use and smoking on the CVD symptom of chest pain using data from Waves 4 and 5 (2016 to 2019). In multivariable models adjusted for covariates, smokers (aOR 1.77; 95% CIs 1.56 to 2.01) and dual‑users (aOR 2.22; 95% CIs 1.61 to 3.05) had higher odds of reporting ever having chest pain, as well as having chest pain in the past 30 days. Conversely, exclusive EVP users had similar odds of reporting chest pain compared to non-users (aOR 1.03; 95% CIs 0.69 to 1.54) and lower odds than smokers and dual‑users. The overall study finding that complete switching to EVP use reduces risk, while continued smoking and dual use do not [186], mirrors findings from other studies.

The All of Us Research Program, a US nationwide longitudinal cohort study [187], has also been used to assess the relationship between EVP use and incident cardiometabolic conditions [188]. Exclusive smoking was found to significantly increase the risk of hypertension, type 2 diabetes mellitus, heart failure and atherosclerotic CVD. Dual use of cigarettes and EVPs also gave rise to a significantly increased risk of hypertension and heart failure and a non‑significant increased risk of type 2 diabetes mellitus and atherosclerotic CVD [188]. Exclusive EVP use was not significantly associated with any of these conditions, with aORs of 1.01 (95% CIs 0.83 to 1.23) for hypertension, 0.88 (95% Cis 0.66 to 11.16) for type 2 diabetes mellitus, 0.82 (95% CIs 0.47 to 1.41) for heart failure, and 1.05 (95% CIs 0.59 to 1.86) for atherosclerotic CVD. These findings can be used to draw two conclusions. Firstly, EVP use is not a risk factor for CVD and related comorbidities. Secondly, assuming that the majority of EVP users are former smokers [159,169], transitioning to EVP use may be protective in terms of reducing CVD risk. 

Population‑level assessments of CVD risk have also been conducted using data collected in the Korean National Health and Nutrition Examination Survey (KNHANES) between 2013 and 2017, although in this study the cohort using EVPs was dual users of those products and cigarettes and formed only a small proportion (approximately 5%) of the overall analysed population [189]. Compared with never smoking, and similar to smoking, dual use was associated with higher waist circumference, elevated blood pressure, fasting glucose and triglycerides, lower HDL cholesterol, and a diagnosis of metabolic syndrome.

Data have also been analysed to assess CVD risk in the Korean National Health Insurance Service survey, in an analysis that utilised a determination of smoking status during a baseline health screening period in 2014/2015 and smoking, HTP or EVP use status at a subsequent follow‑up assessment in 2018 [190]. Those individuals with a prior history of CVD were excluded from the analysis dataset, and CVD was defined as being hospitalised for ≥2 days because of coronary heart disease or stroke. With continuing smokers as a reference, the aHRs for incident cardiovascular disease were 0.83 for dual‑users of cigarettes and novel tobacco/nicotine products, 0.77 for those who had recently (<5 years) quit smoking and using novel products, 0.77 for longer‑term (≥5 years) quitting and novel product use, and 0.55 for never‑smokers [190]. These effects were statistically significant (i.e., the 95% CIs did not span 1) for dual users and recent quitters/novel product users, and the upper 95% CI of 1 for the cohort of longer‑term quitters/novel product users suggests an effect close to significance. This suggests cardiovascular harm reversal associated with quitting smoking and using NGPs.

Furthermore, HRs for quitting without using novel products compared to quitting using novel products were 0.95 and 1.22 for recent and longer‑term cessation, respectively, with the 95% CIs spanning 1 in both cases [190]. This effect was similar when taking pack‑years (a measure of smoking intensity and duration) into consideration. Overall, this similarity in CVD risk among quitters either using or not using NGPs implies a similar risk reduction among those who quit smoking using novel products compared to those who quit unaided or used some other aid for smoking cessation. 

In another Korean assessment of the relationship between HTP use and blood lipid levels using data from the same KNHANES survey between 2018 and 2021, among the HTP‑only users, HDL cholesterol levels were similar, LDL cholesterol was significantly higher, and triglycerides were significantly lower, compared with current smokers [191]. Compared with never smokers, HDL cholesterol levels were significantly lower, and LDL cholesterol and triglycerides were significantly higher, among the HTP‑only users. While these findings do indicate a greater CVD risk, in terms of blood lipid risk factors, among HTP users compared with never smokers/users, it is notable that the statistical models used in the analyses did not adjust for the confounding variable of a prior history of smoking, potentially rendering the findings unreliable. 

The most recent Korean study assessed prognosis after percutaneous coronary intervention (PCI; a clinical procedure used to open coronary arteries narrowed or blocked by atherosclerotic lesions) among individuals in the NHIS survey who were followed up for 3 years following a PCI procedure [192]. For the primary outcome of major adverse cardiac events (MACE), defined as the composite of all‑cause death, spontaneous MI, and repeat revascularisation, multivariable aHRs were 0.82 (95% CIs 0.69 to 0.98) for smokers who switched to EVP use and 0.87 (95% CIs 0.79 to 0.96) among those who had quit all tobacco or nicotine product use. Furthermore, no significant difference was observed in MACE risk between EVP users and non‑smokers/non‑users. Additionally, compared with continued smokers, switching to EVP use led to a reduced risk of all‑cause mortality (aHR 0.64; 95% CIs 0.41 to 0.99), although the risks of spontaneous MI (aHR 0.88; CIs 0.60 to 1.29) or repeat revascularisation (aHR 0.89; 95% CIs 0.73 to 1.07) were not significantly different between continued smokers and those who switched to EVP use. Overall, the study concluded that among smokers who underwent PCI for coronary artery disease, switching to EVP use or quitting smoking was associated with reduced MACE risk compared with continued smoking [192]. 

Japanese data have also been utilised to assess CVD risk among smokers and HTP users. Using data from the Tsuruoka Metabolomics Cohort Study, a prospective cohort study conducted in Tsuruoka City, Harada et al [193] assessed alterations in metabolomic profiles among cigarette smokers and HTP users. In a model adjusted for numerous covariates, HTP use was associated with higher levels of plasma glutamate, a metabolite associated with increased CVD risk [194], compared with never‑smokers, while current smoking was associated with glutamate levels similar to those of HTP use. This study did not, however, control for smoking history among HTP users, which is an important omission given that large numbers of HTP users dual use both cigarettes and HTPs [195]. While the study by Harada et al [193] was limited in scope and assessed only a small Japanese sub‑population, two broader studies by Fujiwara et al [196] and Iwanaga et al [197] have assessed the association between the spread of HTP use and cardiac‑related hospitalisations in the whole Japanese population between 2013 and 2020, using an introduction year of 2017 for HTPs in Japan. In the first study, there were no significant changes in the trends of hospitalisations for acute coronary syndrome, acute MI and unstable angina pectoris before and after January 2017, regardless of seasonal adjustment [196].

However, chronic ischaemic heart disease hospitalisation, chiefly due to elective coronary angiography and PCI, showed a significant increase and reduction before and after January 2017. The authors attributed this not to HTP use but to a change in governmental reimbursement policy aimed at reducing unnecessary PCI procedures in early 2018 [196]. In the second study, which used the same longitudinal trend analysis approach but used data up to 2022, the authors reported a significant association between HTP use and a decreasing trend of acute coronary syndrome hospitalisations among four subgroups in which HTP use was most prevalent (younger adults, smokers, those residing in Tokyo, and those residing in prefectures with high HTP use). While the authors correctly suggest that this is an association, and that further studies are required to assess causation [197], this study suggests that the rising prevalence of HTP use and reductions in cigarette smoking in Japan may be linked with reductions in hospitalisation due to cardiovascular events within that population. 

For EVPs, findings from numerous studies assessing population-level impacts of EVP use compared with cigarette smoking are presented in Table 2. In summary of the findings from these studies and others, it is clear that the exclusive use of both EVPs and HTPs is associated with reduced risk of CVD and related comorbidities compared with continued smoking. Such a conclusion is supported both by cross‑sectional analyses and longitudinal study data from a variety of sources in the US, Japan and Korea. Furthermore, exclusive use of EVPs does not appear to be associated with CVD risk among never-smokers. It is also apparent that dual use of NGPs and cigarettes may convey a similar CVD risk to continued exclusive smoking. In some studies, dual use was associated with a higher CVD risk than exclusive smoking, though this may be due to the confounding factor of higher smoking intensity and therefore toxicant exposure and CVD risk among dual‑users.

**Table 2:** Summary of Population Study Data Examining Associations Between Smoking/EVP Use and CVD Events or Risk Factors. Data are presented as aORs with 95% CIs in parentheses. Bold text denotes significant associations. ^1^Re-analysis of data from [166] when classifying those who had heart attacks before the onset of EVP use as never EVP users. Reference groups were never-smokers and never-users of EVPs. ^2^Reference group was never-smokers. EVP users were never-smokers. ^3^Reference group was cigarette smokers. EVP users were former smokers. ^4^Reference group was non-users. ^5^Reference groups were exclusive smokers (top two rows) or non-users (bottom two rows). ^6^Reference group was never-users of tobacco. ^7^Reference group was non-current use of the respective products. ^8^Reference groups were never-users of the respective products. ^9^Reference groups were current smoker/never-EVP-user for smokers, and never -smoker/never-EVP-users for current EVP users. Current smokers were also EVP users, and current EVP users were never-smokers. ^10^Reference group for current exclusive EVP users was current exclusive smokers. ^11^Reference groups were never-users of the respective products. ^12^Reference groups were never-users of the respective products. ^13^Reference group was never-smokers and never-EVP-users. ^14^Reference groups were non-users of the respective products. ^15^Re-analysis of data from reference [184] when adjusting for various confounders but omitting the ‘age’ variable due to the association between heart attack and EVP use being largely moderated by age. Study population was never-smokers. Reference group was never-smokers. ^16^Reference groups were current smokers (top row) or non-smokers/EVP non-users (bottom row). ^17^Reference groups were non-users of the respective products. ^18^Reference group was continuing smokers. Abbreviations: aOR, adjusted odds ratio; CI, confidence interval; CVD, cardiovascular disease; EVP, electronic vaping product; ND, not determinable due to no incident CVD among exclusive smokers switching to exclusive EVP use; Ref, reference.

Reference	Event/Risk Factor	Smoking	EVP use
[168]^1^	Myocardial infarction	4.25 (2.79-6.47)	0.69 (0.22-2.12)
[169]^2^	Hypertension	1.36 (1.15-1.62)	1.32 (0.50-3.53)
[170]^3^	Interleukin-6	Ref.	0.84 (0.71-0.98)
	High-sensitivity C-reactive protein	Ref.	0.73 (0.57-0.93)
	Fibrinogen	Ref.	0.96 (0.92-1.01)
	Soluble intercellular adhesion molecule-1	Ref.	0.82 (0.75-0.89)
	8-isoprostane	Ref.	0.75 (0.68-0.83)
[171]^4^	High-sensitivity C-reactive protein	1.19 (1.06-1.33)	1.08 (0.92-1.27)
	Interleukin-6	1.15 (1.07-1.23)	1.00 (0.89-1.12)
	Soluble intercellular adhesion molecule-1	1.19 (1.15-1.24)	1.05 (0.99-1.11)
	Fibrinogen	1.04 (1.02-1.06)	1.00 (0.96-1.04)
	8-isoprostane	1.24 (1.15-1.34)	1.02 (0.89-1.17)
[172]^5^	Any cardiovascular disease	Ref.	0.66 (0.46-0.94)
	Myocardial infarction, heart failure or stroke	Ref.	0.61 (0.34-1.09)
	Any cardiovascular disease	1.53 (1.30-1.79)	1.00 (0.69-1.45)
	Myocardial infarction, heart failure or stroke	2.20 (1.73-2.81)	1.35 (0.75-2.42)
[173]^6^	Cardiovascular disease	1.44 (0.87-2.39)	ND
[174]^7^	Myocardial infarction	1.99 (1.40-2.84)	0.61 (0.12-3.04)
	Stroke	2.26 (1.51-3.39)	1.74 (0.55-5.49)
[175]^8^	Stroke	2.1 (1.9-2.4)	1.62 (1.18-2.31)
[176]^9^	Cardiovascular disease	1.36 (1.18-1.56)	1.04 (0.63-1.72)
[177]^10^	Stroke	Ref.	0.43 (0.20-0.33)
[178]^11^	Cardiovascular disease	1.452 (1.332-1.582)	1.170 (0.969-1.412)
[182]^12^	Myocardial infarction	3.13 (2.63-3.73)	1.35 (0.80-2.27)
	Coronary heart disease	1.73 (1.46-2.05)	1.31 (0.79-2.17)
[162]^13^	Myocardial infarction	-	1.65 (0.51-5.32)
[183]^14^	Hypertension	1.384 (1.277-1.499)	1.244 (1.048-1.477)
	Stroke	2.114 (1.815-2.463)	1.058 (0.586-1.911)
	Diabetes mellitus	1.141 (1.023-1.274)	1.108 (0.972-1.263)
	Coronary artery disease	1.862 (1.611-2.151)	0.856 (0.519-1.412)
	Myocardial infarction	2.836 (2.442-3.294)	0.984 (0.555-1.747)
[185]^15^	Myocardial infarction	-	0.80 (0.45-1.43)
	Coronary heart disease	-	0.39 (0.21-0.73)
	Stroke	-	0.44 (0.22-0.88)
[186]^16^	Past 30-day chest pain	Ref.	0.53 (0.32-0.88)
		1.60 (1.34-1.90)	0.85 (0.51-1.41)
[188]^17^	Hypertension	1.20 (1.15-1.26)	1.01 (0.83-1.23)
	Type 2 diabetes mellitus	1.18 (1.11-1.26)	0.88 (0.66-1.16)
	Heart failure	1.50 (1.40-1.62)	0.82 (0.47-1.41)
	Atherosclerotic cardiovascular disease	1.66 (1.51-1.81)	1.05 (0.59-1.86)
[192]^18^	Major adverse cardiovascular event	Ref.	0.82 (0.69-0.98)
	Death from cardiovascular disease	Ref.	0.63 (0.27-1.47)
	Spontaneous myocardial infarction	Ref.	0.88 (0.60-1.29)
	Repeat revascularisation	Ref.	0.89 (0.73-1.07)

As the use of NGPs becomes increasingly prevalent, further studies are required to fully ascertain their CVD risk reduction potential. Importantly, future studies need to fully take into account the smoking history of novel product users, and also to take into account the temporal association between initiation of product use and disease diagnosis to remove the potential for reverse associations being found [165]. Further studies are also required to assess the CVD risk associated with the use of other novel products, such as ONPs. The prevalence of use of ONPs is rising in many countries [198-200], and no studies to date have assessed the CVD risk, in either absolute terms or relative to cigarette smoking, associated with ONP use. However, population‑level studies of tobacco‑containing oral smokeless products, including pouched tobacco (snus), suggest that their use may be associated with reduced toxicant exposure and CVD risk compared to smoking [160,201-205]. While it is reasonable to also suggest that CVD risk associated with ONP use is lower than that of smoking, further studies are required to determine whether or not that is the case. This needs to include an examination of whether, similar to HTP and EVP use, dual use of ONPs and cigarettes is associated with an increased CVD risk. 

Regulatory authority determinations of the reduced CVD risk potential of NGPs

In the US, authorisation to market NGPs falls under the jurisdiction of the US Food and Drug Administration (FDA) following the implementation of the Family Smoking Prevention and Tobacco Control Act in 2009 [206]. The main pathway for authorisation for NGPs is the Premarket Tobacco Product Application (PMTA) pathway [207] and the authorisation of consumer‑facing claims follows that of the Modified Risk Tobacco Product (MRTP) application pathway [208]. The PMTA pathway, simplistically speaking, requires manufacturers to demonstrate that a novel product is ‘Appropriate for the Protection of Public Health’ (APPH) and to consider the risks and benefits to the entire population, including effects on current tobacco product users and non-users (including youth uptake), and to weigh overall whether allowing the product to market would benefit public health. Through the PMTA pathway, marketing authorisations have currently been granted for variants of four brands of EVPs (JUUL, NJOY (Altria, Richmond, USA), Logic (Logic Technology Development LLC, Princeton, USA) and Vuse (R.J. Reynolds Vapor Company, Winston-Salem, USA) [209]) and one brand of ONP (Zyn (Swedish Match AB, Stockholm, Sweden) [210]) as well as for other oral non-tobacco nicotine products (Verve (U.S. Smokeless Tobacco Company/Altria, Richmond, USA) [211]) and for an HTP (IQOS (Philip Morris International, Stamford, USA) [212]). For MRTPs, one modified risk order had been granted for an HTP (IQOS) [213], noting that the authorisation was a modified exposure order and that the information provided by the applicant did not necessarily support a modified risk claim [214]. 

Although the PMTA pathway does not necessitate demonstrating modification of risk-related outcomes, submissions for the products authorised to date have assessed CVD risk. Such assessments have utilised biomarkers in human studies that are either indirectly (BoE to harmful and potentially harmful constituents (HPHCs)) or directly (BoPH) linked to CVD risk, or levels of HPHCs with a known link to CVD [31] in product aerosols and extracts compared with those found in cigarette smoke or traditional oral tobacco products such as snus. This has led, for example, the FDA to conclude that ‘… adults who smoke who switch completely to the new [Zyn ONP] products are expected to experience reduced risk of … cardiovascular toxicity’ [215], a finding based on ONP extract HPHC levels relative to those in cigarette smoke. Similarly, for the Vuse Ciro EVP, the FDA concluded that ‘in terms of carcinogenic and/or cardiovascular/respiratory/reproductive/developmental toxicant HPHCs, the new product aerosols consistently demonstrated reduced potential for overall toxicity compared to cigarette smoke from combusted tobacco comparison products’ [216]. 

In addition to these regulatory determinations, published BoE data have indicated significant reductions in exposure to the CVD‑related HPHCs acrolein, benzene, carbon monoxide and several polycyclic aromatic hydrocarbons (PAH) among adults who smoke who completely switched to using on! ONPs (a product similar in design to the Zyn ONP, made by Helix Innovations, Stockholm, Sweden) [131]. Similar findings have also been reported for the Verve tobacco-free oral nicotine products among those who either partially or completely switched to using Verve [132]. For Vuse Ciro, reductions in exposure to the same CVD‑related HPHCs were reported among adults who smoke and who completely switched to its use [94]. This was associated with significant reductions in the BoPH 2,3‑dinor TXB2, and 11‑dTXB2, markers of platelet activation and aggregation and CVD risk [94,217]. Similar switching studies, as well as cross‑sectional observational studies with the JUUL EVP, have also reported reductions in BoE to CVD-related HPHCs [87,92,140], with some of the reductions similar in magnitude to those seen with complete abstention from nicotine/tobacco product use [87,92]. As for other EVPs, reductions in BoE were associated with lower levels of CVD and inflammatory BoPH sICAM‑1, WBC counts, MCP‑1 and HbA1C among smokers who had switched to using the JUUL EVP compared with continuing smokers [140]. Additionally, higher levels of the cardioprotective HDL cholesterol were also reported among switching smokers [140]. Overall, these BoE and BoPH findings lend support to the FDA determinations of the likelihood of reduced cardiovascular toxicity among smokers who switch to the use of these novel nicotine products.

Regarding the IQOS HTP, which has received marketing orders through both the PMTA and MRTP regulatory pathways [212,213], improvements in CVD risk were featured in both the applications and the FDA decisions. The PMTA and MRTP authorisations noted reductions in HPHCs, as well as in BoE for HPHCs linked to CVD risk, which include acrolein, benzene and carbon monoxide. In some cases, BoE reductions approached levels seen with complete abstinence from tobacco and nicotine product use [212,213]. Furthermore, reductions in BoPH for CVD, inflammation and oxidative stress, including WBC counts, sICAM‑1, 8‑epi‑PGF2α and 11-dTXB2, and an increase in cardioprotective HDL, among smokers switching to using the IQOS HTP were noted [212,213], and favourable changes in hsCRP have also been reported among smokers switching to using IQOS [102,111]. 

Other regulatory authorities have taken a similar approach to allowing modified exposure claims, subject to approval following submission of scientific documentation. This includes Greece, where in 2023 a reduced exposure claim was approved for the IQOS HTP [218] based on reduced exposure to chemical toxicants, including those linked to CVD. This claim was approved under legislation enacted in Greece in 2020 to allow the communication of evidence‑based tobacco product risk messaging and which recognises access to accurate information about regulated products. 

Concluding remarks

Overall, when taking into account data from in vivo and in vitro laboratory studies comparing exposure to aerosols from EVPs or HTPs, or extracts from ONPs, with cigarette smoke exposure, longitudinal and cross‑sectional clinical studies assessing BoE and BoPH among switching smokers, and population studies assessing the impact of NGPs in several countries, NGPs demonstrate strong potential to reduce CVD risk compared with cigarette smoking. Cigarette smoking is a cause of serious diseases, including CVD, and evidence‑based education about NGPs can help reduce the health burden of cigarette smoking both among individual smokers and across populations. It is also important to consider that the level of cigarette consumption influences the degree of CVD risk [1], with even low levels of cigarette consumption significantly increasing the likelihood of CVD development [4,7]. Therefore, incomplete switching and maintaining even a low level of cigarette consumption should be discouraged, and complete abstinence from cigarette smoking should be encouraged. This is supported by several population‑level studies that have found that among dual users of either EVPs or HTPs with cigarettes, CVD risk is similar to that among exclusive cigarette smokers. While dual use may be a transitional step from smoking to exclusive NGP use [219-221], the period of dual use should be minimised in order to provide the greatest CVD risk reduction. 

As the use of NGPs becomes increasingly prevalent in many countries, with these potentially risk-reduced alternatives displacing cigarette smoking, it is important to continually assess the CVD‑related population‑level impact of these products. For example, the number of EVP users in the United Kingdom (UK) has recently been reported to exceed the number of cigarette smokers [222], and this is concurrent with a high prevalence of former smoking. Given the timeframe of CVD risk reversal upon quitting smoking [7-10], such population‑level switching from smoking cigarettes to the use of EVPs may be expected to lead to reductions in CVD diagnoses and hospitalisations in the near future. Evaluating whether such an effect exists is crucial for shaping regulations on NGPs in other countries and for providing smokers with accurate guidance on reducing the potential impact on their cardiovascular health, particularly if quitting tobacco and nicotine use entirely as the best alternative for reducing health risks is not a viable alternative.

It is equally important that studies are undertaken to assess the CVD risk potential, both in absolute terms and relative to cigarette smoking, of ONPs, products that do not contain tobacco leaf and are not used via inhalation. The use prevalence of ONPs is rising in many countries, and due to the nascency of this product category, currently there is a lack of population‑level insight regarding the impact of this on CVD risk. Such studies must be conducted according to the best possible standards of epidemiological assessment, particularly with respect to proper and rigorous control of confounding variables such as former smoking, as well as other risk factors that may be associated with an elevated CVD risk. 

Some regulatory authorities have made determinations that certain NGPs reduce exposure to toxicants with a known link to CVD. These determinations have allowed products to be marketed as reduced exposure products in some countries, but no regulatory authority has, to date, approved any form of modified risk claim. This should not be interpreted as meaning that the use of such products does not pose a lower risk to health compared with cigarette smoking, but that the burden of proof is high and that no manufacturer has met the evidentiary requirements to support a reduced risk claim for a specific NGP. Notably, in this regard and particularly for the population health studies, much of the data described in this review alludes to the reduced CVD risk potential of categories of NGPs and not specific NGPs. Within some product categories, such as EVPs and HTPs, a wide variety of products are available that heat liquids/tobacco by different mechanisms and give rise to different toxicant yields in their aerosols. Further product‑specific data are therefore required to understand the CVD risk reduction potential of individual products within these categories. 

## Conclusions

Overall, when triangulating data from in vitro and in vivo laboratory studies comparing exposure to aerosols/extracts from EVPs, HTPs and ONPs with cigarette smoke exposure, from longitudinal and cross‑sectional clinical studies assessing BoE and BoPH among switching smokers, and from population studies assessing the impact of NGPs in several countries, NGPs demonstrate strong potential to reduce CVD risk compared with cigarette smoking. While this does not mean that the use of NGPs is risk-free, and their use may cause continued dependence on nicotine, our findings suggest that the CVD-related harms of NGP use are significantly lower than those associated with cigarette smoking. Importantly, data supporting this conclusion, obtained from preclinical and clinical laboratory studies, are well-supported by population-level evidence. As the prevalence of NGP use increases in many countries, continuous assessment of the population-level impact of these products, both related to CVD as well as other smoking-related diseases, is imperative. This is particularly the case for ONPs, an emerging category of NGP for which there is a paucity of population-level information concerning changes in CVD risk associated with their use. While some regulatory authorities have made determinations regarding the CVD risk potential of NGPs, others must take into account the ability of NGPs to reduce CVD risk compared with cigarette smoking when setting regulations that may restrict access to NGPs among current smokers. Such restrictions may hamper progress in reducing the global prevalence of CVD. 

## References

[1] (2025). World Health Organization: The Tobacco Atlas. health effects. https://tobaccoatlas.org/challenges/health-effects/.

[2] Fawzy AM, Lip GY (2021). Cardiovascular disease prevention: risk factor modification at the heart of the matter. Lancet Reg Health West Pac.

[3] (2025). Centers for Disease Control and Prevention: health effects of cigarettes: cardiovascular disease. https://www.cdc.gov/tobacco/about/cigarettes-and-cardiovascular-disease.html.

[4] Hackshaw A, Morris JK, Boniface S, Tang JL, Milenković D (2018). Low cigarette consumption and risk of coronary heart disease and stroke: meta-analysis of 141 cohort studies in 55 study reports. BMJ.

[5] Banks E, Joshy G, Korda RJ (2019). Tobacco smoking and risk of 36 cardiovascular disease subtypes: fatal and non-fatal outcomes in a large prospective Australian study. BMC Med.

[6] Roth GA, Mensah GA, Johnson CO (2020). Global burden of cardiovascular diseases and risk factors, 1990-2019: update from the GBD 2019 study. J Am Coll Cardiol.

[7] Tasdighi E, Yao Z, Dardari ZA (2025). Association between cigarette smoking status, intensity, and cessation duration with long-term incidence of nine cardiovascular and mortality outcomes: the Cross-Cohort Collaboration (CCC). PLoS Med.

[8] Rahman M, Alatiqi M, Al Jarallah M, Hussain MY, Monayem A, Panduranga P, Rajan R (2025). Cardiovascular effects of smoking and smoking cessation: a 2024 Update. Glob Heart.

[9] Bolliger CT, Zellweger JP, Danielsson T (2002). Influence of long-term smoking reduction on health risk markers and quality of life. Nicotine Tob Res.

[10] Gallucci G, Tartarone A, Lerose R, Lalinga AV, Capobianco AM (2020). Cardiovascular risk of smoking and benefits of smoking cessation. J Thorac Dis.

[11] Rader DJ, Daugherty A (2008). Translating molecular discoveries into new therapies for atherosclerosis. Nature.

[12] Ross R (1993). The pathogenesis of atherosclerosis: a perspective for the 1990s. Nature.

[13] Ross R (1999). Atherosclerosis--an inflammatory disease. N Engl J Med.

[14] Hadi HA, Carr CS, Al Suwaidi J (2005). Endothelial dysfunction: cardiovascular risk factors, therapy, and outcome. Vasc Health Risk Manag.

[15] Libby P, Ridker PM, Maseri A (2002). Inflammation and atherosclerosis. Circulation.

[16] Cathcart MK (2004). Regulation of superoxide anion production by NADPH oxidase in monocytes/macrophages: contributions to atherosclerosis. Arterioscler Thromb Vasc Biol.

[17] Papaharalambus CA, Griendling KK (2007). Basic mechanisms of oxidative stress and reactive oxygen species in cardiovascular injury. Trends Cardiovasc Med.

[18] Freedman JE (2008). Oxidative stress and platelets. Arterioscler Thromb Vasc Biol.

[19] Galkina E, Ley K (2007). Vascular adhesion molecules in atherosclerosis. Arterioscler Thromb Vasc Biol.

[20] Fearon IM, Faux SP (2009). Oxidative stress and cardiovascular disease: novel tools give (free) radical insight. J Mol Cell Cardiol.

[21] Cai H, Harrison DG (2000). Endothelial dysfunction in cardiovascular diseases: the role of oxidant stress. Circ Res.

[22] Davidson SM, Duchen MR (2007). Endothelial mitochondria: contributing to vascular function and disease. Circ Res.

[23] Madamanchi NR, Runge MS (2007). Mitochondrial dysfunction in atherosclerosis. Circ Res.

[24] Rajagopalan S, Meng XP, Ramasamy S, Harrison DG, Galis ZS (1996). Reactive oxygen species produced by macrophage-derived foam cells regulate the activity of vascular matrix metalloproteinases in vitro. Implications for atherosclerotic plaque stability. J Clin Invest.

[25] Cathcart MK, Chisolm GM 3rd, McNally AK, Morel DW (1988). Oxidative modification of low density lipoprotein (LDL) by activated human monocytes and the cell lines U937 and HL60. In Vitro Cell Dev Biol.

[26] Cathcart MK, Morel DW, Chisolm GM 3rd (1985). Monocytes and neutrophils oxidize low density lipoprotein making it cytotoxic. J Leukoc Biol.

[27] Handin RI, Karabin R, Boxer GJ (1977). Enhancement of platelet function by superoxide anion. J Clin Invest.

[28] Marcus AJ (1979). Pathways of oxygen utilization by stimulated platelets and leukocytes. Semin Hematol.

[29] Baker RR (2006). Smoke generation inside a burning cigarette: modifying combustion to develop cigarettes that may be less hazardous to health. Progress Energ Combust Sci.

[30] Rodgman A, Perfetti T (2013). The Chemical Components of Tobacco and Tobacco Smoke. https://www.routledge.com/The-Chemical-Components-of-Tobacco-and-Tobacco-Smoke/Rodgman-Perfetti/p/book/9781466515482.

[31] (2025). Food and Drug Administration: harmful and potentially harmful constituents in tobacco products and tobacco smoke; established list. https://www.federalregister.gov/documents/2012/04/03/2012-7727/harmful-and-potentially-harmful-constituents-in-tobacco-products-and-tobacco-smoke-established-list.

[32] (2025). Centers for Disease Control and Prevention, National Center for Chronic Disease Prevention and Health Promotion, Office on Smoking and Health: cardiovascular diseases. How tobacco smoke causes disease: the biology and behavioral basis for smoking-attributable disease: a report of the Surgeon General. https://www.ncbi.nlm.nih.gov/books/NBK53017/.

[33] Roy A, Rawal I, Jabbour S, Prabhakaran D (2017). Tobacco and cardiovascular disease: a summary of evidence. Cardiovascular, Respiratory, and Related Disorders. Disease Control Priorities (Third Edition).

[34] Makena P, Haswell LE, McEwan M (2025). An adverse outcome pathway for cigarette smoke-mediated oxidative stress in plaque formation. Front Toxicol.

[35] Rhee MY, Na SH, Kim YK, Lee MM, Kim HY (2007). Acute effects of cigarette smoking on arterial stiffness and blood pressure in male smokers with hypertension. Am J Hypertens.

[36] Benowitz NL, Fraiman JB (2017). Cardiovascular effects of electronic cigarettes. Nat Rev Cardiol.

[37] Jukic I, Becic T, Matulic I, Simac P, Vukovic J (2025). Impact of nicotine-free electronic cigarettes on cardiovascular health: a systematic review. J Clin Med.

[38] McNeil A, Brose LS, Calder R, Bauld L, Robson D (2018). Evidence Review of E-Cigarettes and Heated Tobacco Products 2018.

[39] Royal College of Physicians (2016). Nicotine without Smoke: Tobacco Harm Reduction - A Report by the Tobacco Advisory Group of the Royal College of Physicians. https://www.rcp.ac.uk/media/xcfal4ed/nicotine-without-smoke_0.pdf.

[40] Cordery S, Thompson K, Stevenson M (2024). The product science of electrically heated tobacco products: an updated narrative review of the scientific literature. Cureus.

[41] Cao Y, Zhang L, Yang M (2025). Assessing biomarkers of exposure to carcinogens associated with combustible cigarettes, electronic cigarettes, and heated tobacco products: a systematic review and meta-analysis. Front Pharmacol.

[42] Institute of Medicine (2001). Clearing the Smoke: Assessing the Science Base for Tobacco Harm Reduction. Clearing the Smoke: Assessing the Science Base for Tobacco Harm Reduction.

[43] Stratton K, Shetty P, Wallace R, Bondurant S (2001). Clearing the smoke: the science base for tobacco harm reduction--executive summary. Tob Control.

[44] National Academies of Sciences E (2018). Medicine: Public Health Consequences of E-Cigarettes. Public Health Consequences of E-Cigarettes: 2018.

[45] (2025). World Health Organization: The Tobacco Atlas. E-cigarettes and HTPs. https://tobaccoatlas.org/challenges/e-cigarettes-htps/.

[46] Grandolfo E, Ogden H, Fearon IM, Malt L, Stevenson M, Weaver S, Nahde T (2024). Tobacco-free nicotine pouches and their potential contribution to tobacco harm reduction: a scoping review. Cureus.

[47] Travis N, Warner KE, Goniewicz ML (2025). The potential impact of oral nicotine pouches on public health: a scoping review. Nicotine Tob Res.

[48] Kouzoukas E, Navas C, Zawertailo L (2026). The health effects of vaping and e-cigarettes: consensus recommendations. Int J Drug Policy.

[49] Fearon IM, Gaça MD, Nordskog BK (2013). In vitro models for assessing the potential cardiovascular disease risk associated with cigarette smoking. Toxicol In Vitro.

[50] Chen HW, Lii CK, Ku HJ, Wang TS (2009). Cigarette smoke extract induces expression of cell adhesion molecules in HUVEC via actin filament reorganization. Environ Mol Mutagen.

[51] Halvorsen B, Lund Sagen E, Ueland T, Aukrust P, Tonstad S (2007). Effect of smoking cessation on markers of inflammation and endothelial cell activation among individuals with high risk for cardiovascular disease. Scand J Clin Lab Invest.

[52] Reichert V, Xue X, Bartscherer D (2009). A pilot study to examine the effects of smoking cessation on serum markers of inflammation in women at risk for cardiovascular disease. Chest.

[53] Taylor M, Jaunky T, Hewitt K, Breheny D, Lowe F, Fearon IM, Gaca M (2017). A comparative assessment of e-cigarette aerosols and cigarette smoke on in vitro endothelial cell migration. Toxicol Lett.

[54] Farsalinos KE, Romagna G, Allifranchini E (2013). Comparison of the cytotoxic potential of cigarette smoke and electronic cigarette vapour extract on cultured myocardial cells. Int J Environ Res Public Health.

[55] Simms L, Yu F, Palmer J (2022). Use of human induced pluripotent stem cell-derived cardiomyocytes to predict the cardiotoxicity potential of next generation nicotine products. Front Toxicol.

[56] Su L, Zhao M, Ma F (2022). A comparative assessment of e-cigarette aerosol extracts and tobacco cigarette smoke extracts on in vitro endothelial cell inflammation response. Hum Exp Toxicol.

[57] Chapman F, Sticken ET, Wieczorek R (2023). Multiple endpoint in vitro toxicity assessment of a prototype heated tobacco product indicates substantially reduced effects compared to those of combustible cigarette. Toxicol In Vitro.

[58] Bishop E, Breheny D, Hewitt K (2020). Evaluation of a high-throughput in vitro endothelial cell migration assay for the assessment of nicotine and tobacco delivery products. Toxicol Lett.

[59] Simms L, Mason E, Berg EL (2021). Use of a rapid human primary cell-based disease screening model, to compare next generation products to combustible cigarettes. Curr Res Toxicol.

[60] Makwana O, Smith GA, Flockton HE, Watters GP, Lowe F, Breheny D (2021). Impact of cigarette versus electronic cigarette aerosol conditioned media on aortic endothelial cells in a microfluidic cardiovascular model. Sci Rep.

[61] Chapman F, de Haan L, Gijzen L (2024). Optimisation of an in vitro human cardiovascular model on-a-chip for toxicological assessment of nicotine delivery products. Front Toxicol.

[62] Ohashi K, Hayashida A, Nozawa A, Matsumura K, Ito S (2023). Human vasculature-on-a-chip with macrophage-mediated endothelial activation: the biological effect of aerosol from heated tobacco products on monocyte adhesion. Toxicol In Vitro.

[63] Giebe S, Hofmann A, Brux M, Lowe F, Breheny D, Morawietz H, Brunssen C (2021). Comparative study of the effects of cigarette smoke versus next generation tobacco and nicotine product extracts on endothelial function. Redox Biol.

[64] Poussin C, Kramer B, Lanz HL (2020). 3D human microvessel-on-a-chip model for studying monocyte-to-endothelium adhesion under flow - application in systems toxicology. ALTEX.

[65] Poussin C, Laurent A, Peitsch MC, Hoeng J, De Leon H (2016). Systems toxicology-based assessment of the candidate modified risk tobacco product THS2.2 for the adhesion of monocytic cells to human coronary arterial endothelial cells. Toxicology.

[66] Poussin C, Laurent A, Kondylis A (2018). In vitro systems toxicology-based assessment of the potential modified risk tobacco product CHTP 1.2 for vascular inflammation- and cytotoxicity-associated mechanisms promoting adhesion of monocytic cells to human coronary arterial endothelial cells. Food Chem Toxicol.

[67] Poussin C, van der Toorn M, Scheuner S (2021). Systems toxicology study reveals reduced impact of heated tobacco product aerosol extract relative to cigarette smoke on premature aging and exacerbation effects in aged aortic cells in vitro. Arch Toxicol.

[68] Hayashida A, Nozawa A, Ito S (2025). Monocyte migration assay using a vascular-on-a-chip model and its utilization for the evaluation of a heated tobacco product. Front Toxicol.

[69] Phillips B, Veljkovic E, Boué S (2016). An 8-month systems toxicology inhalation/cessation study in Apoe-/- mice to investigate cardiovascular and respiratory exposure effects of a candidate modified risk tobacco product, THS 2.2, compared with conventional cigarettes. Toxicol Sci.

[70] Ilyas I, Little PJ, Liu Z (2022). Mouse models of atherosclerosis in translational research. Trends Pharmacol Sci.

[71] Golforoush P, Yellon DM, Davidson SM (2020). Mouse models of atherosclerosis and their suitability for the study of myocardial infarction. Basic Res Cardiol.

[72] Phillips B, Szostak J, Titz B (2019). A six-month systems toxicology inhalation/cessation study in ApoE(-/-) mice to investigate cardiovascular and respiratory exposure effects of modified risk tobacco products, CHTP 1.2 and THS 2.2, compared with conventional cigarettes. Food Chem Toxicol.

[73] Szostak J, Titz B, Schlage WK (2020). Structural, functional, and molecular impact on the cardiovascular system in ApoE(-/-) mice exposed to aerosol from candidate modified risk tobacco products, Carbon Heated Tobacco Product 1.2 and Tobacco Heating System 2.2, compared with cigarette smoke. Chem Biol Interact.

[74] Szostak J, Wong ET, Titz B (2020). A 6-month systems toxicology inhalation study in ApoE(-/-) mice demonstrates reduced cardiovascular effects of E-vapor aerosols compared with cigarette smoke. Am J Physiol Heart Circ Physiol.

[75] Wong ET, Szostak J, Titz B (2021). A 6-month inhalation toxicology study in Apoe(-/-) mice demonstrates substantially lower effects of e-vapor aerosol compared with cigarette smoke in the respiratory tract. Arch Toxicol.

[76] Lao CJ, Jordan MC, Rivera JC (2025). Conventional tobacco and electronic cigarettes differentially affect cardiovascular health in male and female mice. Am J Physiol Heart Circ Physiol.

[77] Asrafuzzaman M, Cao Y, Afroz R, Kamato D, Gray S, Little PJ (2017). Animal models for assessing the impact of natural products on the aetiology and metabolic pathophysiology of Type 2 diabetes. Biomed Pharmacother.

[78] Olfert IM, DeVallance E, Hoskinson H (2018). Chronic exposure to electronic cigarettes results in impaired cardiovascular function in mice. J Appl Physiol (1985).

[79] Wong ET, Luettich K, Krishnan S (2020). Reduced chronic toxicity and carcinogenicity in A/J mice in response to life-time exposure to aerosol from a heated tobacco product compared with cigarette smoke. Toxicol Sci.

[80] (2026). Organisation for Economic Co-operation and Development: guidance document for the scientific review of adverse outcome pathways. https://www.oecd.org/content/dam/oecd/en/publications/reports/2021/12/guidance-document-for-the-scientific-review-of-adverse-outcome-pathways_07c6ef3f/a6bec14b-en.pdf..

[81] Ding R, Ren X, Sun Q, Sun Z, Duan J (2023). An integral perspective of canonical cigarette and e-cigarette-related cardiovascular toxicity based on the adverse outcome pathway framework. J Adv Res.

[82] Yuki D, Takeshige Y, Nakaya K, Futamura Y (2018). Assessment of the exposure to harmful and potentially harmful constituents in healthy Japanese smokers using a novel tobacco vapor product compared with conventional cigarettes and smoking abstinence. Regul Toxicol Pharmacol.

[83] Round EK, Chen P, Taylor AK, Schmidt E (2019). Biomarkers of tobacco exposure decrease after smokers switch to an e-cigarette or nicotine gum. Nicotine Tob Res.

[84] Pulvers K, Nollen NL, Rice M, Schmid CH, Qu K, Benowitz NL, Ahluwalia JS (2020). Effect of pod e-cigarettes vs cigarettes on carcinogen exposure among African American and Latinx smokers: a randomized clinical trial. JAMA Netw Open.

[85] McRobbie H, Phillips A, Goniewicz ML, Smith KM, Knight-West O, Przulj D, Hajek P (2015). Effects of switching to electronic cigarettes with and without concurrent smoking on exposure to nicotine, carbon monoxide, and acrolein. Cancer Prev Res (Phila).

[86] Arnold MJ, Nollen NL, Mayo MS (2021). Harm reduction associated with dual use of cigarettes and e-cigarettes in black and latino smokers: secondary analyses from a randomized controlled e-cigarette switching trial. Nicotine Tob Res.

[87] Cohen G, Goldenson NI, Bailey PC, Chan S, Shiffman S (2021). Changes in biomarkers of cigarette smoke exposure after 6 days of switching exclusively or partially to use of the JUUL system with two nicotine concentrations: a randomized controlled confinement study in adult smokers. Nicotine Tob Res.

[88] Cravo AS, Bush J, Sharma G, Savioz R, Martin C, Craige S, Walele T (2016). A randomised, parallel group study to evaluate the safety profile of an electronic vapour product over 12 weeks. Regul Toxicol Pharmacol.

[89] D'Ruiz CD, Graff DW, Robinson E (2016). Reductions in biomarkers of exposure, impacts on smoking urge and assessment of product use and tolerability in adult smokers following partial or complete substitution of cigarettes with electronic cigarettes. BMC Public Health.

[90] Edmiston JS, Webb KM, Wang J, Oliveri D, Liang Q, Sarkar M (2022). Biomarkers of exposure and biomarkers of potential harm in adult smokers who switch to e-vapor products relative to cigarette smoking in a 24-week, randomized, clinical trial. Nicotine Tob Res.

[91] Goniewicz ML, Gawron M, Smith DM, Peng M, Jacob P 3rd, Benowitz NL (2017). Exposure to nicotine and selected toxicants in cigarette smokers who switched to electronic cigarettes: a longitudinal within-subjects observational study. Nicotine Tob Res.

[92] Jay J, Pfaunmiller EL, Huang NJ, Cohen G, Graff DW (2020). Five-day changes in biomarkers of exposure among adult smokers after completely switching from combustible cigarettes to a nicotine-salt pod system. Nicotine Tob Res.

[93] Kanobe MN, Jones BA, Nelson P (2022). Part three: a randomized study to assess biomarker changes in cigarette smokers switched to Vuse Solo or Abstinence. Sci Rep.

[94] Kanobe MN, Nelson PR, Brown BG (2023). Changes in biomarkers of exposure and potential harm in smokers switched to Vuse Vibe or Vuse Ciro electronic nicotine delivery systems. Toxics.

[95] Li C, Guo Y, Duan K (2024). Changes in biomarkers of exposure and withdrawal symptom among Chinese adult smokers after completely or partially switching from combustible cigarettes to an electronic nicotine delivery system. Intern Emerg Med.

[96] McEwan M, Gale N, Ebajemito JK, Camacho OM, Hardie G, Proctor CJ, Murphy J (2021). A randomized controlled study in healthy participants to explore the exposure continuum when smokers switch to a tobacco heating product or an E-cigarette relative to cessation. Toxicol Rep.

[97] Morris P, McDermott S, Chapman F (2022). Reductions in biomarkers of exposure to selected harmful and potentially harmful constituents following exclusive and partial switching from combustible cigarettes to myblu(™) electronic nicotine delivery systems (ENDS). Intern Emerg Med.

[98] Walele T, Bush J, Koch A, Savioz R, Martin C, O'Connell G (2018). Evaluation of the safety profile of an electronic vapour product used for two years by smokers in a real-life setting. Regul Toxicol Pharmacol.

[99] Dai H, Benowitz NL, Achutan C, Farazi PA, Degarege A, Khan AS (2022). Exposure to toxicants associated with use and transitions between cigarettes, e-cigarettes, and no tobacco. JAMA Netw Open.

[100] O'Connell G, Graff DW, D'Ruiz CD (2016). Reductions in biomarkers of exposure (BoE) to harmful or potentially harmful constituents (HPHCs) following partial or complete substitution of cigarettes with electronic cigarettes in adult smokers. Toxicol Mech Methods.

[101] Chu S, Li X, Zhang D (2025). Impact of heating conventional cigarettes with a novel device on health-related biomarkers and cigarette use patterns among Chinese adult smokers unwilling to quit: a pilot randomized controlled trial. Nicotine Tob Res.

[102] Ansari SM, Hession PS, David M, Blanc N, de La Bourdonnaye G, Pouly S, Haziza C (2024). Impact of switching from cigarette smoking to tobacco heating system use on biomarkers of potential harm in a randomized trial. Biomarkers.

[103] Li X, Wang X, Cui P, Liu G, Zhang H, Gao Y, Kai Z (2023). Comparison of biomarkers of exposure in a controlled study of smokers switched from conventional cigarettes to heated tobacco products. Toxics.

[104] Nishihara D, Yuki D, Suzuki T, Sakaguchi C, Nagata Y, Kakehi A (2024). A randomized control study in healthy adult smokers to assess reduced exposure to selected cigarette smoke constituents in switching to the novel heated tobacco product DT3.0a. Clin Pharmacol Drug Dev.

[105] Yuki D, Kikuchi A, Suzuki T, Sakaguchi C, Huangfu D, Nagata Y, Kakehi A (2022). Assessment of the exposure to selected smoke constituents in adult smokers using in-market heated tobacco products: a randomized, controlled study. Sci Rep.

[106] Gale N, McEwan M, Hardie G, Proctor CJ, Murphy J (2022). Changes in biomarkers of exposure and biomarkers of potential harm after 360 days in smokers who either continue to smoke, switch to a tobacco heating product or quit smoking. Intern Emerg Med.

[107] Gale N, McEwan M, Camacho OM, Hardie G, Proctor CJ, Murphy J (2021). Changes in biomarkers after 180 days of tobacco heating product use: a randomised trial. Intern Emerg Med.

[108] Gale N, McEwan M, Camacho OM, Hardie G, Murphy J, Proctor CJ (2021). Changes in biomarkers of exposure on switching from a conventional cigarette to the glo tobacco heating product: a randomized, controlled ambulatory study. Nicotine Tob Res.

[109] Bosilkovska M, Tran CT, de La Bourdonnaye G, Taranu B, Benzimra M, Haziza C (2020). Exposure to harmful and potentially harmful constituents decreased in smokers switching to carbon-heated tobacco product. Toxicol Lett.

[110] Lüdicke F, Ansari SM, Lama N (2019). Effects of switching to a heat-not-burn tobacco product on biologically relevant biomarkers to assess a candidate modified risk tobacco product: a randomized trial. Cancer Epidemiol Biomarkers Prev.

[111] Haziza C, de La Bourdonnaye G, Donelli A (2020). Favorable changes in biomarkers of potential harm to reduce the adverse health effects of smoking in smokers switching to the Menthol Tobacco Heating System 2.2 for 3 months (Part 2). Nicotine Tob Res.

[112] Haziza C, de La Bourdonnaye G, Donelli A (2020). Reduction in exposure to selected harmful and potentially harmful constituents approaching those observed upon smoking abstinence in smokers switching to the Menthol Tobacco Heating System 2.2 for 3 months (part 1). Nicotine Tob Res.

[113] Gale N, McEwan M, Eldridge AC (2019). Changes in biomarkers of exposure on switching from a conventional cigarette to tobacco heating products: a randomized, controlled study in healthy Japanese subjects. Nicotine Tob Res.

[114] Lüdicke F, Picavet P, Baker G, Haziza C, Poux V, Lama N, Weitkunat R (2018). Effects of switching to the Menthol Tobacco Heating System 2.2, smoking abstinence, or continued cigarette smoking on clinically relevant risk markers: a randomized, controlled, open-label, multicenter study in sequential confinement and ambulatory settings (part 2). Nicotine Tob Res.

[115] Lüdicke F, Picavet P, Baker G, Haziza C, Poux V, Lama N, Weitkunat R (2018). Effects of switching to the Tobacco Heating System 2.2 Menthol, smoking abstinence, or continued cigarette smoking on biomarkers of exposure: a randomized, controlled, open-label, multicenter study in sequential confinement and ambulatory settings (part 1). Nicotine Tob Res.

[116] Haziza C, de La Bourdonnaye G, Skiada D, Ancerewicz J, Baker G, Picavet P, Lüdicke F (2017). Biomarker of exposure level data set in smokers switching from conventional cigarettes to Tobacco Heating System 2.2, continuing smoking or abstaining from smoking for 5 days. Data Brief.

[117] Martin F, Talikka M, Ivanov NV, Haziza C, Hoeng J, Peitsch MC (2016). Evaluation of the Tobacco Heating System 2.2. Part 9: application of systems pharmacology to identify exposure response markers in peripheral blood of smokers switching to THS2.2. Regul Toxicol Pharmacol.

[118] Haziza C, de La Bourdonnaye G, Skiada D, Ancerewicz J, Baker G, Picavet P, Lüdicke F (2016). Evaluation of the Tobacco Heating System 2.2. Part 8: 5-day randomized reduced exposure clinical study in Poland. Regul Toxicol Pharmacol.

[119] Haziza C, de La Bourdonnaye G, Merlet S (2016). Assessment of the reduction in levels of exposure to harmful and potentially harmful constituents in Japanese subjects using a novel tobacco heating system compared with conventional cigarettes and smoking abstinence: a randomized controlled study in confinement. Regul Toxicol Pharmacol.

[120] Lüdicke F, Baker G, Magnette J, Picavet P, Weitkunat R (2017). Reduced exposure to harmful and potentially harmful smoke constituents with the Tobacco Heating System 2.1. Nicotine Tob Res.

[121] Lüdicke F, Haziza C, Weitkunat R, Magnette J (2016). Evaluation of biomarkers of exposure in smokers switching to a carbon-heated tobacco product: a controlled, randomized, open-label 5-day exposure study. Nicotine Tob Res.

[122] Ogden MW, Marano KM, Jones BA, Morgan WT, Stiles MF (2015). Switching from usual brand cigarettes to a tobacco-heating cigarette or snus: part 2. Biomarkers of exposure. Biomarkers.

[123] Sakaguchi C, Kakehi A, Minami N, Kikuchi A, Futamura Y (2014). Exposure evaluation of adult male Japanese smokers switched to a heated cigarette in a controlled clinical setting. Regul Toxicol Pharmacol.

[124] Martin Leroy C, Jarus-Dziedzic K, Ancerewicz J, Lindner D, Kulesza A, Magnette J (2012). Reduced exposure evaluation of an Electrically Heated Cigarette Smoking System. Part 7: a one-month, randomized, ambulatory, controlled clinical study in Poland. Regul Toxicol Pharmacol.

[125] Tricker AR, Kanada S, Takada K, Martin Leroy C, Lindner D, Schorp MK, Dempsey R (2012). Reduced exposure evaluation of an Electrically Heated Cigarette Smoking System. Part 6: 6-day randomized clinical trial of a menthol cigarette in Japan. Regul Toxicol Pharmacol.

[126] Tricker AR, Jang IJ, Martin Leroy C, Lindner D, Dempsey R (2012). Reduced exposure evaluation of an Electrically Heated Cigarette Smoking System. Part 4: eight-day randomized clinical trial in Korea. Regul Toxicol Pharmacol.

[127] Tricker AR, Kanada S, Takada K, Leroy CM, Lindner D, Schorp MK, Dempsey R (2012). Reduced exposure evaluation of an Electrically Heated Cigarette Smoking System. Part 5: 8-day randomized clinical trial in Japan. Regul Toxicol Pharmacol.

[128] Tricker AR, Stewart AJ, Leroy CM, Lindner D, Schorp MK, Dempsey R (2012). Reduced exposure evaluation of an Electrically Heated Cigarette Smoking System. Part 3: eight-day randomized clinical trial in the UK. Regul Toxicol Pharmacol.

[129] Frost-Pineda K, Zedler BK, Oliveri D, Feng S, Liang Q, Roethig HJ (2008). Short-term clinical exposure evaluation of a third-generation electrically heated cigarette smoking system (EHCSS) in adult smokers. Regul Toxicol Pharmacol.

[130] Frost-Pineda K, Zedler BK, Oliveri D, Liang Q, Feng S, Roethig HJ (2008). 12-week clinical exposure evaluation of a third-generation electrically heated cigarette smoking system (EHCSS) in adult smokers. Regul Toxicol Pharmacol.

[131] Rensch J, Edmiston J, Wang J, Jin X, Sarkar M (2023). A randomized, controlled study to assess changes in biomarkers of exposures among adults who smoke that switch to oral nicotine pouch products relative to continuing smoking or stopping all tobacco use. J Clin Pharmacol.

[132] Edmiston J, Liu J, Wang J, Sarkar M (2022). A randomized, controlled study to assess biomarkers of exposure in adult smokers switching to oral nicotine products. J Clin Pharmacol.

[133] Krautter GR, Borgerding MF (2014). Comparison of consumption patterns, biomarkers of exposure, and subjective effects in cigarette smokers who switched to dissolvable tobacco (Camel Orbs), dual use, or tobacco abstinence. Nicotine Tob Res.

[134] Goldenson NI, Shiffman S, Oliveri D, Liang Q, Black RA (2025). Changes in exposure to tobacco-related harmful and potentially harmful constituents among adults who switched completely from smoking cigarettes to use of the JUUL2 system for six days. Biomarkers.

[135] Cunningham A, McAdam K, Thissen J, Digard H (2020). The evolving e-cigarette: comparative chemical analyses of e-cigarette vapor and cigarette smoke. Front Toxicol.

[136] Li X, Luo Y, Jiang X (2019). Chemical analysis and simulated pyrolysis of Tobacco Heating System 2.2 compared to conventional cigarettes. Nicotine Tob Res.

[137] Werley MS, Freelin SA, Wrenn SE (2008). Smoke chemistry, in vitro and in vivo toxicology evaluations of the electrically heated cigarette smoking system series K. Regul Toxicol Pharmacol.

[138] Ardati O, Adeniji A, El Hage R (2024). Impact of smoking intensity and device cleaning on IQOS emissions: comparison with an array of cigarettes. Tob Control.

[139] Ansari SM, Leroy P, de La Bourdonnaye G, Pouly S, Reese L, Haziza C (2025). Differences in biomarkers of potential harm after 2+ years of tobacco heating system use compared to cigarette smoking: a cross-sectional study. Biomarkers.

[140] Shiffman S, Oliveri DR, Goldenson NI, Liang Q, Black RA, Mishra S (2024). Comparing adult smokers who switched to JUUL versus continuing smokers: biomarkers of exposure and of potential harm and respiratory symptoms. Nicotine Tob Res.

[141] Oliveri D, Liang Q, Sarkar M (2020). Real-world evidence of differences in biomarkers of exposure to select harmful and potentially harmful constituents and biomarkers of potential harm between adult e-vapor users and adult cigarette smokers. Nicotine Tob Res.

[142] Ussher M, Lewis S, Marczylo T (2025). Toxicant and nicotine exposure in pregnant smokers, vapers, and nicotine-replacement users: cross-sectional study. Nicotine Tob Res.

[143] Haswell LE, Gale N, Brown E (2023). Biomarkers of exposure and potential harm in exclusive users of electronic cigarettes and current, former, and never smokers. Intern Emerg Med.

[144] Goniewicz ML, Smith DM, Edwards KC (2018). Comparison of nicotine and toxicant exposure in users of electronic cigarettes and combustible cigarettes. JAMA Netw Open.

[145] Shahab L, Goniewicz ML, Blount BC (2017). Nicotine, carcinogen, and toxin exposure in long-term e-cigarette and nicotine replacement therapy users: a cross-sectional study. Ann Intern Med.

[146] Azzopardi D, Haswell LE, Frosina J (2023). Assessment of biomarkers of exposure and potential harm, and physiological and subjective health measures in exclusive users of nicotine pouches and current, former and never smokers. Biomarkers.

[147] Roethig HJ, Feng S, Liang Q, Liu J, Rees WA, Zedler BK (2008). A 12-month, randomized, controlled study to evaluate exposure and cardiovascular risk factors in adult smokers switching from conventional cigarettes to a second-generation electrically heated cigarette smoking system. J Clin Pharmacol.

[148] Ogden MW, Marano KM, Jones BA, Morgan WT, Stiles MF (2015). Switching from usual brand cigarettes to a tobacco-heating cigarette or snus: Part 3. Biomarkers of biological effect. Biomarkers.

[149] Scherer G (2018). Suitability of biomarkers of biological effects (BOBEs) for assessing the likelihood of reducing the tobacco related disease risk by new and innovative tobacco products: a literature review. Regul Toxicol Pharmacol.

[150] D'Ruiz CD, O'Connell G, Graff DW, Yan XS (2017). Measurement of cardiovascular and pulmonary function endpoints and other physiological effects following partial or complete substitution of cigarettes with electronic cigarettes in adult smokers. Regul Toxicol Pharmacol.

[151] Carnevale R, Sciarretta S, Violi F (2016). Acute impact of tobacco vs electronic cigarette smoking on oxidative stress and vascular function. Chest.

[152] Biondi-Zoccai G, Sciarretta S, Bullen C (2019). Acute effects of heat-not-burn, electronic vaping, and traditional tobacco combustion cigarettes: the Sapienza University of Rome-vascular assessment of proatherosclerotic effects of smoking ( SUR - VAPES ) 2 randomized trial. J Am Heart Assoc.

[153] George J, Hussain M, Vadiveloo T (2019). Cardiovascular effects of switching from tobacco cigarettes to electronic cigarettes. J Am Coll Cardiol.

[154] Świątkowska B, Jankowski M, Kaleta D (2025). Effects of heated tobacco use on blood parameters and cardiovascular risk in healthy men. Med Sci Monit.

[155] Kelesidis T, Fotooh Abadi L, Ruedisueli I, D'Costa ZU, Middlekauff HR (2025). Atherogenic effects of acute electronic cigarette compared with tobacco cigarette smoking in people living with HIV: a randomized crossover trial. J Am Heart Assoc.

[156] Ruedisueli I, Shi K, Lopez S, Gornbein J, Middlekauff HR (2024). Arrhythmogenic effects of acute electronic cigarette compared to tobacco cigarette smoking in people living with HIV. Physiol Rep.

[157] Nguyen R, Ruedisueli I, Lakhani K, Ma J, Middlekauff HR (2024). Acute cardiovascular effects of 4th generation electronic cigarettes and combusted cigarettes: implications for harm reduction. J Appl Physiol (1985).

[158] Lee J, Yao Z, Boakye E, Blaha MJ (2024). The impact of chronic electronic cigarette use on endothelial dysfunction measured by flow-mediated vasodilation: a systematic review and meta-analysis. Tob Induc Dis.

[159] Tarran R, Barr RG, Benowitz NL (2021). E-cigarettes and cardiopulmonary health. Function (Oxf).

[160] Benowitz NL (2025). Predicting cardiovascular risk with use of various tobacco products. Circulation.

[161] Hyland A, Ambrose BK, Conway KP (2017). Design and methods of the Population Assessment of Tobacco and Health (PATH) study. Tob Control.

[162] Critcher CR, Siegel M (2021). Re-examining the association between e-cigarette use and myocardial infarction: a cautionary tale. Am J Prev Med.

[163] Pérez-Guerrero EE, Guillén-Medina MR, Márquez-Sandoval F (2024). Methodological and statistical considerations for cross-sectional, case-control, and cohort studies. J Clin Med.

[164] Savitz DA, Wellenius GA (2023). Can cross-sectional studies contribute to causal inference? It depends. Am J Epidemiol.

[165] Rodu B, Plurphanswat N (2023). Cross-sectional e-cigarette studies are unreliable without timing of exposure and disease diagnosis. Intern Emerg Med.

[166] Bhatta DN, Glantz SA (2019). Electronic cigarette use and myocardial infarction among adults in the US population assessment of tobacco and health. J Am Heart Assoc.

[167] (2020). Retraction to: electronic cigarette use and myocardial infarction among adults in the US population assessment of tobacco and health. J Am Heart Assoc.

[168] Rodu B, Plurphanswat N (2020). A re-analysis of e-cigarette use and heart attacks in PATH wave 1 data. Addiction.

[169] Miller CR, Shi H, Li D, Goniewicz ML (2021). Cross-sectional associations of smoking and e-cigarette use with self-reported diagnosed hypertension: findings from Wave 3 of the Population Assessment of Tobacco and Health Study. Toxics.

[170] Christensen CH, Chang JT, Rostron BL (2021). Biomarkers of inflammation and oxidative stress among adult former smoker, current e-cigarette users-results from Wave 1 PATH study. Cancer Epidemiol Biomarkers Prev.

[171] Stokes AC, Xie W, Wilson AE (2021). Association of cigarette and electronic cigarette use patterns with levels of inflammatory and oxidative stress biomarkers among US adults: Population Assessment of Tobacco and Health study. Circulation.

[172] Berlowitz JB, Xie W, Harlow AF (2022). E-cigarette use and risk of cardiovascular disease: a longitudinal analysis of the PATH study (2013-2019). Circulation.

[173] Mahoney MC, Rivard C, Kimmel HL (2022). Cardiovascular outcomes among combustible-tobacco and Electronic Nicotine Delivery System (ENDS) users in Waves 1 through 5 of the Population Assessment of Tobacco and Health (PATH) study, 2013-2019. Int J Environ Res Public Health.

[174] Hirschtick JL, Cook S, Patel A (2023). Longitudinal associations between exclusive and dual use of electronic nicotine delivery systems and cigarettes and self-reported incident diagnosed cardiovascular disease among adults. Nicotine Tob Res.

[175] Bricknell RA, Ducaud C, Figueroa A (2021). An association between electronic nicotine delivery systems use and a history of stroke using the 2016 behavioral risk factor surveillance system. Medicine (Baltimore).

[176] Osei AD, Mirbolouk M, Orimoloye OA (2019). Association between e-cigarette use and cardiovascular disease among never and current combustible-cigarette smokers. Am J Med.

[177] Parekh T, Pemmasani S, Desai R (2020). Risk of stroke with e-cigarette and combustible cigarette use in young adults. Am J Prev Med.

[178] Liu X, Yuan Z, Ji Y (2022). The association between electronic cigarettes, sleep duration, and the adverse cardiovascular outcomes: findings from behavioral risk factor surveillance system, 2020. Front Cardiovasc Med.

[179] Wang JB, Olgin JE, Nah G, Vittinghoff E, Cataldo JK, Pletcher MJ, Marcus GM (2018). Cigarette and e-cigarette dual use and risk of cardiopulmonary symptoms in the Health eHeart Study. PLoS One.

[180] Patel U, Patel N, Khurana M (2022). RETRACTED: effect comparison of e-cigarette and traditional smoking and association with stroke-a cross-sectional study of NHANES. Neurol Int.

[181] Patel U, Patel N, Khurana M (2025). RETRACTED: Patel et al. effect comparison of e-cigarette and traditional smoking and association with stroke-a cross-sectional study of NHANES. Neurol. Int. 2022, 14, 441-452. Neurol Int.

[182] Farsalinos KE, Polosa R, Cibella F, Niaura R (2019). Is e-cigarette use associated with coronary heart disease and myocardial infarction? Insights from the 2016 and 2017 National Health Interview Surveys. Ther Adv Chronic Dis.

[183] Falk GE, Okut H, Vindhyal MR, Ablah E (2022). Hypertension and cardiovascular diseases among electronic and combustible cigarette users. Kans J Med.

[184] Alzahrani T (2023). Electronic cigarette use and myocardial infarction. Cureus.

[185] Plurphanswat N, Selya A, Rodu B (2024). Questionable effects of electronic cigarette use on cardiovascular diseases from the national health interview survey (NHIS, 2014-2021). Cureus.

[186] Behrooz L, Xie W, Goghari A, Robertson R, Bhatnagar A, Stokes A, Hamburg NM (2024). Electronic cigarette use and chest pain in US adults: evidence from the PATH study. Tob Induc Dis.

[187] Denny JC, Rutter JL, Goldstein DB, Philippakis A, Smoller JW, Jenkins G, Dishman E (2019). The "All of Us" research program. N Engl J Med.

[188] Erhabor J, Yao Z, Tasdighi E, Benjamin EJ, Bhatnagar A, Blaha MJ (2025). E-cigarette use and incident cardiometabolic conditions in the all of us research program. Nicotine Tob Res.

[189] Kim CY, Paek YJ, Seo HG (2020). Dual use of electronic and conventional cigarettes is associated with higher cardiovascular risk factors in Korean men. Sci Rep.

[190] Choi S, Lee K, Park SM (2021). Combined associations of changes in noncombustible nicotine or tobacco product and combustible cigarette use habits with subsequent short-term cardiovascular disease risk among South Korean men: a nationwide cohort study. Circulation.

[191] Kim M, Lee KJ, Choi I, Kim SH, Ryu K (2025). Relationship between heated tobacco product use and low-density lipoprotein cholesterol in Korean adults: a cross-sectional study using Korea National Health and Nutrition Examination Survey 2018-2021 (VII-1 and VIII). Korean J Fam Med.

[192] Kang D, Choi KH, Kim H (2025). Prognosis after switching to electronic cigarettes following percutaneous coronary intervention: a Korean nationwide study. Eur Heart J.

[193] Harada S, Ohmomo H, Matsumoto M (2024). Metabolomics profiles alterations in cigarette smokers and heated tobacco product users. J Epidemiol.

[194] Zheng Y, Hu FB, Ruiz-Canela M (2016). Metabolites of glutamate metabolism are associated with incident cardiovascular events in the PREDIMED PREvención con DIeta MEDiterránea (PREDIMED) trial. J Am Heart Assoc.

[195] Fearon IM, Cordery SF, Fitzpatrick M (2024). A scoping review of behavioural studies on heated tobacco products. Cureus.

[196] Fujiwara H, Nakai M, Iwanaga Y (2023). No significant change in trend of hospitalizations for acute coronary syndrome in japan before and after introduction of heated tobacco products. Circ Cardiovasc Qual Outcomes.

[197] Iwanaga Y, Nakai M, Miyamoto Y, Hirano T, Fujiwara H (2025). Heated tobacco product spread and hospitalizations for acute coronary syndrome in Japan. JAMA Netw Open.

[198] Brose L, Bunce L, Cheeseman H (2026). Prevalence of nicotine pouch use among youth and adults in great britain-analysis of cross-sectional, nationally representative surveys. Nicotine Tob Res.

[199] Felicione NJ, Ozga JE, Eversole A (2026). Oral nicotine pouches: rising popularity and state of the science. Public Health Rep.

[200] Jankowski M, Rees VW (2024). Awareness and use of nicotine pouches in a nationwide sample of adults in Poland. Tob Induc Dis.

[201] Rezk-Hanna M, Warda US, Stokes AC (2022). Associations of smokeless tobacco use with cardiovascular disease risk: insights from the population assessment of tobacco and health study. Nicotine Tob Res.

[202] Yao Z, Tasdighi E, Dardari ZA (2025). Differential associations of cigar, pipe, and smokeless tobacco use versus combustible cigarette use with subclinical markers of inflammation, thrombosis, and atherosclerosis: the Cross-Cohort Collaboration-Tobacco Working Group. Circulation.

[203] Clarke E, Thompson K, Weaver S, Thompson J, O'Connell G (2019). Snus: a compelling harm reduction alternative to cigarettes. Harm Reduct J.

[204] Lee PN (2013). Epidemiological evidence relating snus to health--an updated review based on recent publications. Harm Reduct J.

[205] Lee PN (2013). The effect on health of switching from cigarettes to snus - a review. Regul Toxicol Pharmacol.

[206] (2025). Food and Drug Administration: family smoking prevention and tobacco control act - an overview. https://www.fda.gov/tobacco-products/rules-regulations-and-guidance-related-tobacco-products/family-smoking-prevention-and-tobacco-control-act-overview.

[207] (2025). Food and Drug Administration: premarket tobacco product applications. https://www.fda.gov/tobacco-products/market-and-distribute-tobacco-product/premarket-tobacco-product-applications..

[208] (2025). Food and Drug Administration: modified risk tobacco products. https://www.fda.gov/tobacco-products/advertising-and-promotion/modified-risk-tobacco-products..

[209] (2025). Food and Drug Administration: e-cigarettes authorized by the FDA. https://digitalmedia.hhs.gov/tobacco/hosted/Authorized-E-Cig-July2025.pdf.

[210] (2025). Food and Drug Administration: FDA authorizes marketing of 20 ZYN nicotine pouch products after extensive scientific review. https://www.fda.gov/news-events/press-announcements/fda-authorizes-marketing-20-zyn-nicotine-pouch-products-after-extensive-scientific-review..

[211] (2025). Food and Drug Administration: PMTA scientific review: technical project lead (TPL). https://www.fda.gov/media/153255/download.

[212] (2025). Food and Drug Administration: PMTA coversheet: technical project lead review (TPL). https://www.accessdata.fda.gov/static/searchtobacco/2019/ctp-callahan-pmta-tpl-043019_19_0.pdf.

[213] (2025). Food and Drug Administration: scientific review of modified risk tobacco product application (MRTPA) under section 911(d) of the FD&C Act -technical project lead. https://www.fda.gov/media/139796/download.

[214] Food and Drug Administration: Philip Morris Products S.A (2025). Food and Drug Administration: Philip Morris Products S.A. modified risk tobacco product (MRTP) applications. https://www.fda.gov/tobacco-products/advertising-and-promotion/philip-morris-products-sa-modified-risk-tobacco-product-mrtp-applications.

[215] (2025). Food and Drug Administration: technical project lead review of PMTAs. https://www.accessdata.fda.gov/static/searchtobacco/ZYN/PMTA_TPL_PM593-PM612_Zyn_01_13_2025_Redacted.pdf.

[216] (2025). Food and Drug Administration: technical project lead (TPL) review of PMTAs. https://www.accessdata.fda.gov/static/searchtobacco/2022/RJRV-TPL-MultipleSTNs.pdf..

[217] Makena P, Liu G, Chen P, Yates CR, Prasad GL (2019). Urinary leukotriene E(4) and 2,3-dinor thromboxane B(2) are biomarkers of potential harm in short-term tobacco switching studies. Cancer Epidemiol Biomarkers Prev.

[218] (2025). Global Institute for Novel Nicotine: Greece sets a European precedent with science-based regulation of smoke-free products. https://www.ginn.global/greece-sets-a-european-precedent-with-science-based-regulation-of-smoke-free-products/.

[219] Li L, Borland R, Cummings KM (2021). Patterns of non-cigarette tobacco and nicotine use among current cigarette smokers and recent quitters: findings from the 2020 ITC four country smoking and vaping survey. Nicotine Tob Res.

[220] Pesola F, Myers Smith K, Przulj D, Ladmore D, Phillips-Waller A, McRobbie H, Hajek P (2026). Patterns of e-cigarette use and smoking cessation outcomes: secondary analysis of a large randomised controlled trial to inform clinical advice. Nicotine Tob Res.

[221] Sugiyama T, Tabuchi T (2020). Use of multiple tobacco and tobacco-like products including heated tobacco and e-cigarettes in Japan: a cross-sectional assessment of the 2017 JASTIS study. Int J Environ Res Public Health.

[222] Iacobucci G (2025). Vaping overtakes smoking in Britain for first time. BMJ.

